# DNA replication stress underpins the vulnerability to oxidative phosphorylation inhibition in colorectal cancer

**DOI:** 10.1038/s41419-025-07334-4

**Published:** 2025-01-14

**Authors:** Xiao Hong Zhao, Man Man Han, Qian Qian Yan, Yi Meng Yue, Kaihong Ye, Yuan Yuan Zhang, Liu Teng, Liang Xu, Xiao-Jing Shi, Ting La, Yu Chen Feng, Ran Xu, Vinod K. Narayana, David P. De Souza, Lake-Ee Quek, Jeff Holst, Tao Liu, Mark A. Baker, Rick F. Thorne, Xu Dong Zhang, Lei Jin

**Affiliations:** 1 School of Biomedical Sciences and Pharmacy, The University of Newcastle, Newcastle, NSW Australia; 2 Tianjian Laboratory of Advanced Biomedical Sciences, Academy of Medical Sciences, Zhengzhou University, Zhengzhou, Henan China; 3 Translational Research Institute, Henan Provincial and Zhengzhou City Key Laboratory of Noncoding RNA and Cancer Metabolism, Henan International Join Laboratory of Noncoding RNA and Metabolism in Cancer, Henan Provincial People’s Hospital, Zhengzhou University, Zhengzhou, Henan China; 4 National-Local Joint Engineering Research Center of Biodiagnosis & Biotherapy, The Second Affiliated Hospital, Xi’an Jiaotong University, Xi’an, Shaanxi China; 5 School of Medicine and Public Health, The University of Newcastle, Newcastle, NSW Australia; 6 Bio21 Institute and Department of Biochemistry and Molecular Biology, University of Melbourne, Parkville, VIC Australia; 7 Metabolomics Australia, University of Melbourne, Parkville, VIC Australia; 8 School of Mathematics and Statistics, The University of Sydney, Sydney, NSW Australia; 9 School of Biomedical Sciences, University of New South Wales, Sydney, NSW Australia; 10 Children’s Cancer Institute Australia for Medical Research, University of New South Wales, Sydney, NSW Australia

**Keywords:** Cancer metabolism, Colon cancer, Metabolomics

## Abstract

Mitochondrial oxidative phosphorylation (OXPHOS) is a therapeutic vulnerability in glycolysis-deficient cancers. Here we show that inhibiting OXPHOS similarly suppresses the proliferation and tumorigenicity of glycolytically competent colorectal cancer (CRC) cells in vitro and in patient-derived CRC xenografts. While the increased glycolytic activity rapidly replenished the ATP pool, it did not restore the reduced production of aspartate upon OXPHOS inhibition. This shortage in aspartate, in turn, caused nucleotide deficiencies, leading to S phase cell cycle arrest, replication fork stalling, and enrichment of the p53 pathway, manifestations of replication stress. The addition of purine nucleobases adenine and guanine along with the pyrimidine nucleoside uridine restored replication fork progression and cell proliferation, whereas the supplementation of exogenous aspartate recovered the nucleotide pool, demonstrating a causal role of the aspartate shortage in OXPHOS inhibition-induced nucleotide deficiencies and consequently replication stress and reductions in proliferation. Moreover, we demonstrate that glutamic-oxaloacetic transaminase 1 (GOT1) is critical for maintaining the minimum aspartate pool when OXPHOS is inhibited, as knockdown of GOT1 further reduced aspartate levels and rendered CRC cells more sensitive to OXPHOS inhibition both in vitro and in vivo. These results propose GOT1 targeting as a potential avenue to sensitize cancer cells to OXPHOS inhibitors, thus lowering the necessary doses to efficiently inhibit cancer growth while alleviating their adverse effects.

## Introduction

Cancer cells frequently boost mitochondrial oxidative phosphorylation (OXPHOS) to fulfill their heightened energy and biosynthesis needs [1, 2], making the inhibition of mitochondrial complex I a promising approach for cancer treatment [3–6]. However, recent clinical studies have revealed that dose-limiting toxicities prevent patients from receiving higher doses of the inhibitors, thus precluding their effectiveness as anti-cancer drugs [7]. Consequently, strategies are needed to provide therapeutic benefits at clinically achievable doses.

Cancer cell metabolism is highly heterogenous: along with increased OXPHOS, the Warburg effect, i.e., aerobic glycolysis is also commonly activated [8, 9]. Moreover, cancer cell metabolism is flexible with the Warburg effect and OXPHOS readily rewireable to allow for their rapid adaptation to the often-adverse microenvironment [10]. In fact, the Warburg effect or OXPHOS prevails in a cancer type- and context-dependent manner [10]. For example, acute myeloid leukemia (AML) cells heavily rely on OXPHOS, whereas colorectal cancer (CRC) cells commonly exhibit the glycolytic phenotype [3, 11]. Furthermore, certain cancer cells become deficient in glycolysis and addicted to OXPHOS due to specific genetic and epigenetic anomalies [3, 4, 12].

Glycolysis-deficient cancer cells are hypersensitive to targeting OXPHOS [3, 13], mirrored by the great potency of mitochondrial complex I inhibitors to constrain their proliferation with or without induction of cell death in preclinical studies [3, 6]. However, the phenotypical and mechanistic consequences of targeting OXPHOS in glycolysis-competent cancer cells remains less understood. While increased glycolysis may fully replenish energy, it cannot entirely restore the impaired biosynthesis upon OXPHOS inhibition [14–17]. This implicates that targeting OXPHOS may also be effective in glycolysis-competent cancers. Indeed, mitochondrion-depleted cells are auxotrophic for exogenous uridine [16], implicating the importance of nucleotide homeostasis supported by OXPHOS. Nonetheless, the role of interruptions to biosynthesis in determining the responses of glycolysis-competent cancer cells to targeting OXPHOS has not been unequivocally established.

Here we demonstrate that targeting OXPHOS suppresses proliferation and tumorigenicity in glycolysis-competent CRC cells, similar to glycolysis-deficient cancer cells. The mechanism involves DNA replication stress caused by nucleotide deficiencies resulting from a shortage in the nucleobase precursor, aspartate. Moreover, we show that the cytoplasmic enzyme glutamic-oxaloacetic transaminase 1 (GOT1) plays an important role in replenishing the aspartate pool upon OXPHOS inhibition. These results suggest that targeting OXPHOS is potentially effective in the treatment of CRCs and other types of cancers that are capable of glycolysis, with the inhibition of GOT1 as a potential approach to sensitize CRC cells to OXPHOS-targeting therapies.

## Materials and methods

### Cell culture and human tissues

The human CRC cell lines Colo205, Caco-2, LS411N, SW620, RKO, HCT116 were from American Type Culture Collection (ATCC) as described previously [18]. The human CRC cell line LIM1215 was from the European Collection of Authenticated Cell Cultures (ECACC). The human neuroblastoma cell line NB-1 was from CellBank Australia. All cell lines were cultured in DMEM (Gibco, #11960044) supplemented with 5% fetal bovine serum (FBS, #SFBS; Bovogen, Australia), 4 mM glutamine (Gibco, #25030149) and 8 µg/ml Gentamicin (Gibco, #15710-064). All cell lines were verified to be free of mycoplasma contamination using PCR every 3 months and were authenticated using short tandem repeat profiling by the Australian Genome Research Facility. Studies using patient colorectal cancer tumor tissues were approved by the Human Research Ethics Committee of the Henan Provincial People’s Hospital (2023-48; Zhengzhou, China).

### Antibodies and reagents

Information on antibodies and reagents used in this study is provided in Tables S1 and S2, respectively.

### Small interference RNA (siRNA) and short hairpin RNA (shRNA)

SiRNAs were obtained from GenePharma (Shanghai, China) and transfected using the lipofectamine 3000 Transfection Kit (ThermoFisher Scientific, #L3000-075) as previously described [18]. ShRNA oligos were purchased from Integrated DNA Technologies (Coralville, IA) and cell sub-lines carrying the inducible shRNA system in response to doxycycline (Dox) were established as described previously [19]. The siRNA and shRNA sequences are listed in Table S3.

### Cell viability

Cell viability was measured using the Fluorometric-Blue Cell Viability Kit (#ab102501, Abcam) according to the manufacturer’s instructions as described previously [18].

### Apoptosis

Apoptotic cells were quantitated using the FITC Annexin V Apoptosis Detection Kit (#556547, BD Biosciences) according to the manufacturer’s instructions as described previously [18].

### Mouse models

Studies on animals were conducted in accordance with relevant guidelines and regulations and were approved by the Animal Research Ethics Committee of the University of Newcastle (A-2021-121; Newcastle, Australia) and Animal Laboratory Center of Zhengzhou University (ZZU-LAC20230526; Zhengzhou, China). Additional details are in supplemental methods.

### Colorimetric assays and other detailed experimental procedures

Detailed experimental methods about cell cycle, plasmids, RNA sequencing, ECAR and OCR measurements, Cellular ATP, lactate, aspartate, ROS level measurements, glucose and glutamine consumption and extracellular lactate assays, mitochondrial complex I and II activity measurements, DNA fiber assays, western blotting, IHC, targeted metabolomics, stable isotope tracing, xenograft mouse models and purification of CRC cells from tumor xenografts are provided in the Supplementary Material and Methods section.

### Statistical analysis

Statistical analysis was carried out using GraphPad Prism 10 to assess differences between experimental groups. Statistical differences were analyzed by two-tailed Student’s *t* test, one-way ANOVA test followed by Tukey’s multiple comparisons test. *P* values lower than 0.05 were considered statistically significant.

## Results

### OXPHOS inhibition triggers S phase cell cycle arrest in glycolysis-competent CRC cells

To test the effect of targeting OXPHOS on glycolysis-competent cancer cells, we assembled a panel of CRC cell lines with defined genotypes that possess the intact glycolytic machinery as manifested by their basal glycolytic activities and capacities, measured using ECAR assays (Fig. S1A and Table S4). As a control, we included the neuroblastoma cell line NB-1, which is defective in glycolysis due to the loss of phosphogluconate dehydrogenase [3]. Treatment with the mitochondrial complex I inhibitor IACS-010759 markedly reduced the OCR in the CRC and NB-1 cells (Fig. S1B, C) [3], substantiating the inhibition of OXPHOS. Concurrently, the ECAR was increased in the CRC cells (Fig. S1D, E), indicating a metabolic switch towards glycolysis. Further corroborating its specificity, IACS-010759 treatment markedly reduced mitochondrial complex I but not complex II activity (Fig. S1F, G).

**Fig. 1:**
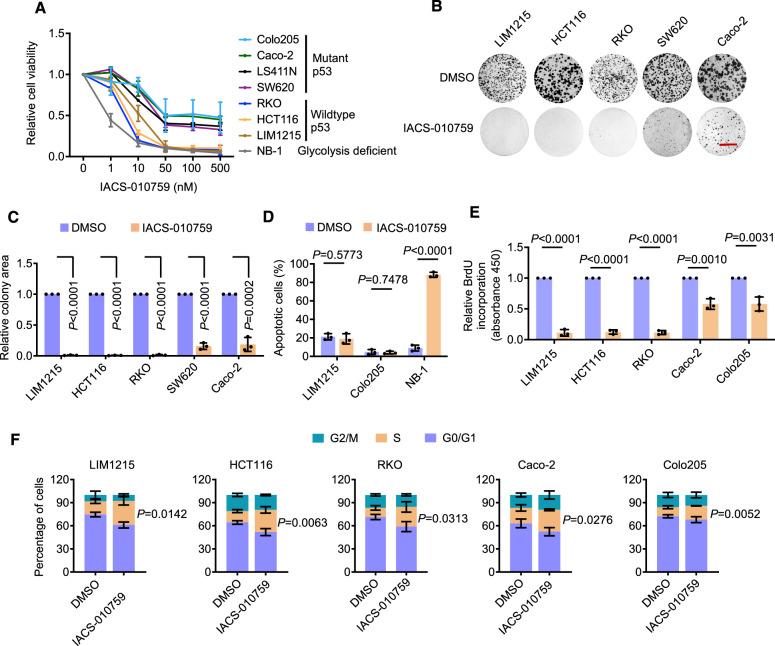
OXPHOS inhibition induces S phase cell cycle arrest in glycolysis-competent CRC cells. **A** CRC cells and NB-1 neuroblastoma cells were treated with IACS-010759 at indicated concentrations for 72 h before cell viability assays. The relative cell viability before treatment was arbitrarily designated as 1. Data shown are mean ± S.D. of results from 3 individual experiments. **B**, **C** CRC cells treated with the vehicle control (DMSO) or IACS-010759 (100 nM) were subjected to clonogenic assays (**B**), with relative clonogenicity quantified using ImageJ-plugin “ColonyArea” (**C**). The relative clonogenicity of cells treated DMSO was arbitrarily designated as 1 (**C**). Data shown are representatives (**B**) or mean ± S.D. (**C**) of results from 3 individual experiments, two-tailed Student’s *t* test. Scale bar: 1 cm. **D** LIM1215, Colo205 CRC cells, and NB-1 neuroblastoma cells, were treated with DMSO or IACS-010759 (100 nM) for 72 h before apoptosis was quantified. Data shown are mean ± S.D. of results from 3 individual experiments, two-tailed Student’s *t* test. **E** CRC cells treated with DMSO or IACS-010759 (100 nM) for 72 h were subjected to BrdU incorporation assays. The relative BrdU incorporation in cells before treatment was arbitrarily designated as 1. Data shown are mean ± S.D. of results from 3 individual experiments, two-tailed Student’s *t* test. **F** CRC cells treated with DMSO or IACS-010759 (100 nM) for 72 h were subjected to cell cycle analysis. Data shown are mean ± S.D. of results from 3 individual experiments, two-tailed Student’s *t* test.

IACS-010759 treatment inhibited the viability to varying degrees in these glycolysis-competent CRC cell lines, with notable sensitivity in cells carrying wildtype p53 (RKO, HCT116, LIM1215) compared to mutant p53 counterparts (Colo205, Caco-2, LS411N, SW620), similar to NB-1 cells (Fig. 1A). Moreover, wildtype p53 CRC cells also displayed higher sensitivity to the other complex I inhibitor IM156 than those harboring mutant p53 (Fig. S1H), as measured using the cell viability assay. On the other hand, there was no significant relationship between the sensitivity and the mutational status in APC, KRAS, and BRAF, as well as genomic amplification of MYC—common genetic anomalies in CRCs (Fig. 1A and Table S4) [20]. Similarly, there was no discernible association between the sensitivity and the relative activity of glycolysis to OXPHOS (Fig. S1I). Decreased cell viability was echoed by decreases in clonogenicity in CRC cells treated with IACS-010759 (Fig. 1B, C). Nevertheless, the difference in sensitivity between wildtype and mutant p53 cells diminished in the clonogenic assay (Fig. 1B, C). Together, these results indicate that CRC cells are commonly susceptible to OXPHOS inhibition. Moreover, they suggest that while p53 might influence the response, it may not play a major role in determining the ultimate fate of the cells under this stress.

Although IACS-010759 treatment killed NB-1 cells, as reported previously [3], it did not induce significant cell death in LIM1215 and Colo205 cells (Fig. 1D). This indicates that cancer cells capable of glycolysis can survive OXPHOS inhibition at the cost of reduced proliferation. Indeed, IACS-010759 treatment reduced BrdU incorporation and triggered cell cycle arrest at S phase (Fig. 1E, F). Similarly, treatment with IM156 also caused S phase cell cycle arrest in CRC cells (Fig. S1J). Taken together, these results suggest that inhibiting proliferation through cell cycle arrest at S phase is the predominant functional consequence of targeting OXPHOS in glycolysis-competent CRC cells.

### OXPHOS inhibition retards CRC growth in vivo

**Fig. 2:**
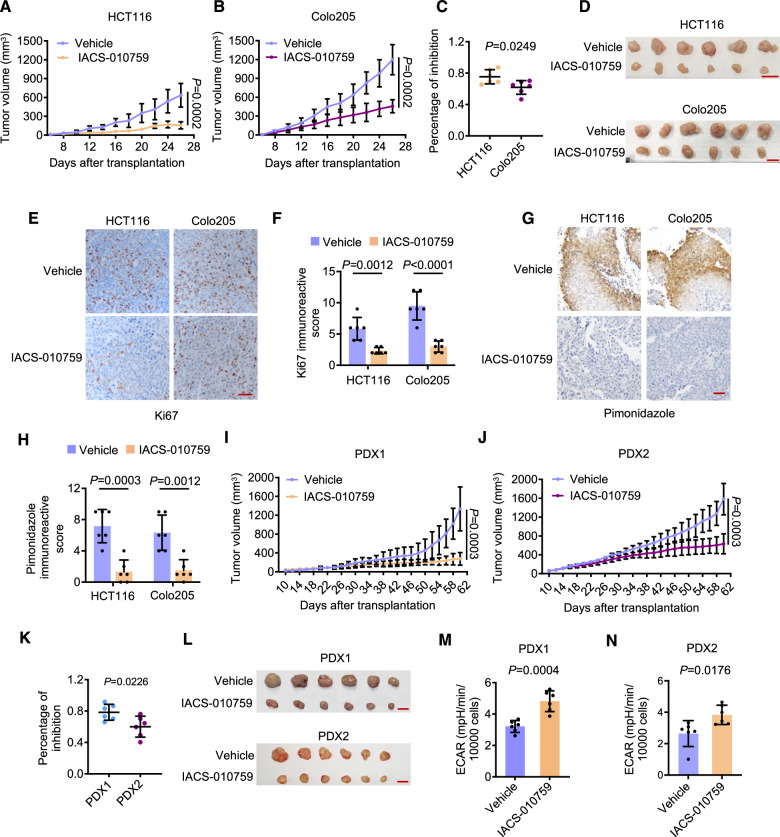
OXPHOS inhibition retards CRC growth in vivo. **A**–**C** Growth curves of HCT116 (**A**) and Colo205 (**B**) xenografts treated with the vehicle (0.5% methyl cellulose) or IACS-010759 (15 mg/kg), and comparison of the potencies of IACS-010759 treatment in the xenografts (**C**). Data shown are mean ± S.D.; n = 6 mice per group, two-tailed Student’s *t* test. **D** Photographs of the HCT116 and Colo205 xenografts treated as in (**A**, **B**). Scale bar: 1 cm. **E**, **F** Representative microphotographs (**E**) and quantification (**F**) of IHC staining of Ki67 on FFPE sections of HCT116 and Colo205 xenografts treated as in (**A**, **B**). Data shown are representatives or mean ± S.D.; n = 6 mice per group, two-tailed Student’s *t* test. Scale bar: 50 μm. **G**, **H** Representative microphotographs (**G**) and quantification (**H**) of IHC staining of pimonidazole on FFPE sections of HCT116 and Colo205 xenografts treated as in (**A**, **B**). Data shown are representatives or mean ± S.D.; n = 6 mice per group, two-tailed Student’s *t* test. Scale bar: 50 μm. **I-K** Growth curves of PDX1 (**I**) and PDX2 (**J**) tumors treated with the vehicle (0.5% methyl cellulose) or IACS-010759 (15 mg/kg), and comparison of the potencies of IACS-010759 treatment in the PDXs (**K**). Data shown are mean ± S.D.; n = 6 mice per group, two-tailed Student’s *t* test. **L** Photographs of the PDX1 and PDX2 treated as in (**I**, **J**). Scale bar: 1 cm. **M**, **N** CRC cells isolated from PDX1 (**M**) and PDX2 (**N**) treated as in (**I**, **J**) were subjected to measurement of the ECAR. Data shown are mean ± S.D.; n = 6 mice per group, two-tailed Student’s *t* test.

Consistent with the findings in vitro (Fig. 1A), IACS-010759 treatment reduced HCT116 and Colo205 xenograft growth in NSG mice, with HCT116 tumors (wildtype p53) more sensitive, albeit moderately, than those derived from Colo205 cells (mutant p53) (Figs. 2A–D and S2A). The inhibition of tumorigenicity by IACS-010759 treatment was associated with decreases in the proportion of Ki67-positive CRC cells (Fig. 2E, F), indicative of reduced cell proliferation. Moreover, the fraction of CRC cells positive for exogenous pimonidazole, which can bind to thiol groups of proteins under hypoxic conditions [3], decreased (Fig. 2G, H), demonstrating that microenvironmental hypoxia was mitigated, conceivably due to the reduced oxygen consumption following treatment with IACS-010759 [12].

To further investigate the therapeutic potential of targeting OXPHOS in CRC, we employed two independent patient-derived xenograft (PDX) models established in NOD/SCID mice with treatment-naïve CRCs that exhibited the intact glycolytic machinery (Fig. S2B). While PDX1 carried wildtype p53, PDX2 harbored an inactive mutant of p53 (c.637 C > T). IACS-010759 treatment significantly inhibited the growth of the PDXs, with PDX1 displaying noticeably higher sensitivity than PDX2 (Figs. 2I–L and S2C). The inhibition of PDX growth by IACS-010759 treatment was similarly associated with decreases in the proportion of Ki67-positive CRC cells, and reductions in the fraction of CRC cells positive for pimonidazole (Fig. S2D–G). Importantly, CRC cells isolated from PDX1 and PDX2 in mice treated with IACS-010759 exhibited increased ECAR compared to those from mice treated with the vehicle control (Fig. 2M, N), indicating enhanced glycolytic activity. Collectively, these results suggest that targeting OXPHOS is potentially effective for the treatment of CRCs.

### OXPHOS inhibition does not cause devastating energy stress in glycolysis-competent CRC cells

**Fig. 3:**
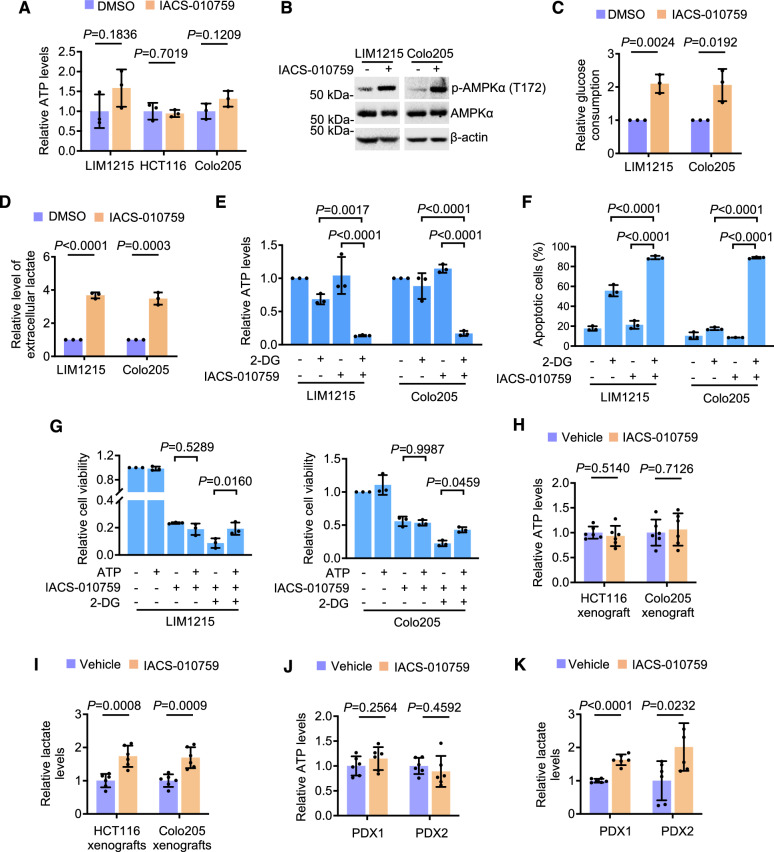
OXPHOS inhibition does not cause devastating energy stress in glycolysis-competent CRC cells. **A** CRC cells treated with the DMSO or IACS-010759 (100 nM) for 24 h were subjected to ATP measurement using LC-MS analysis. Data shown are mean ± S.D. of results from 3 technique repeats, two-tailed Student’s *t* test. **B** LIM1215 and Colo205 cells treated with the DMSO or IACS-010759 (100 nM) for 15 min were subjected to Western blot analysis. Data shown are representatives of 3 individual experiments. **C**, **D** LIM1215 and Colo205 cells were treated with the DMSO or IACS-010759 (100 nM) for 24 h. The culture media were subjected to colorimetric glucose consumption assays (**C**) and colorimetric lactate assays (**D**). Data shown are mean ± S.D. of results from 3 individual experiments, two-tailed Student’s *t* test. **E**, **F** LIM1215 and Colo205 cells treated as indicated for 24 h were subjected to colorimetric ATP assays (**E**) and apoptosis assay (**F**). IACS-010759, 100 nM; 2-DG, 50 mM. Data shown are mean ± S.D. of results from 3 individual experiments, two-tailed Student’s *t* test. **G** LIM1215 and Colo205 treated as indicated for 72 h were subjected to cell viability assays. ATP, 100 μΜ; IACS-010759, 100 nM; 2-DG, 50 mM. Data shown are mean ± S.D. of results from 3 individual experiments, one-way ANOVA followed by Tukey’s multiple comparison test. **H**, **I** CRC cells isolated from HCT116 and Colo205 xenografts in mice treated with the vehicle or IACS-010759 (15 mg/kg) were subjected to colorimetric ATP (**H**) or lactate assays (**I**). Data shown are mean ± S.D.; n = 6 mice per group, two-tailed Student’s *t* test. **J**, **K** CRC cells isolated from PDX1 and PDX2 tumors in mice treated as in (**H**, **I**) were subjected to colorimetric ATP (**J**) or lactate assays (**K**). Data shown are mean ± S.D., n = 6 mice per group, two-tailed Student’s *t* test.

We focused on investigating the mechanisms responsible for the inhibition of proliferation caused by targeting OXPHOS in CRC cells. Intriguingly, cellular ATP levels in LIM1215, HCT116, and Colo205 cells after exposure to IACS-010759 were not reduced, as shown in LC-MS analysis (Fig. 3A). This was further confirmed using luminescent ATP assays (Fig. S3A). Similarly, the levels of ATP were not decreased in CRC cells treated with IM156 (Fig. S3B). In contrast, ATP was depleted in NB-1 cells after exposure to IACS-010759 or IM156 (Fig. S3A, B). These results, along with the increases in glycolysis (Fig. S1D, E), demonstrated that the cellular ATP pools were rapidly refilled by the increased glycolytic activity in CRC cells upon OXPHOS inhibition. In support, exposure to IACS-010759 caused a swift increase in the activation of 5′ AMP-activated protein kinase (AMPK), an essential sensor of energy deficiencies that initiates cellular adaptation to energy stress [21], in LIM1215 and Colo205 cells (Fig. 3B). Moreover, glucose consumption and extracellular lactate levels were markedly increased, contrary to the reduced consumption of glutamine, in the cells after IACS-010759 treatment (Figs. 3C, D and S3C).

To test the importance of increased glycolysis in the response of CRC cells to targeting OXPHOS, we co-treated LIM1215 and Colo205 cells with the glycolysis inhibitor 2-deoxy-d-glucose (2-DG) and IACS-010759. This co-treatment led to cellular ATP depletion and marked cell death (Fig. 3E, F). Adding exogenous ATP to the culture medium to replenish ATP levels partially rescued cells from the co-treatment [22], but it did not affect the reductions in cell viability caused by IACS-010759 alone (Fig. 3G). These results support the idea that increased glycolysis is crucial for CRC survival, and OXPHOS inhibition doesn’t reduce cell proliferation due to an energy crisis. Consistently, CRC cells isolated from HCT116 and Colo205 xenografts as well as PDX1 and PDX2 from mice treated with IACS-010759 displayed similar ATP levels and increased levels of cellular lactate compared with corresponding controls (Fig. 3H–K), substantiating that CRC cells can retain adequate ATP production through increased glycolysis when OXPHOS is inhibited in vivo.

### OXPHOS inhibition induces DNA replication stress in CRC cells

**Fig. 4:**
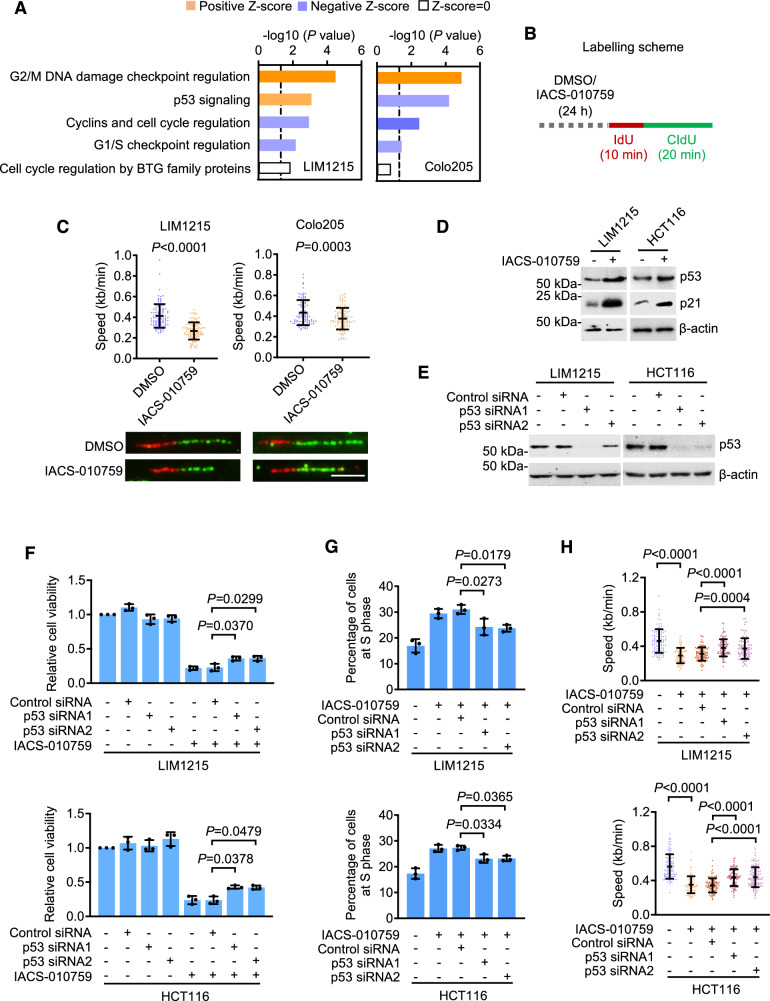
OXPHOS inhibition induces DNA replication stress in CRC cells. **A** IPA of RNA-seq data from LIM1215 and Colo205 cells treated with DMSO or IACS-010759 (100 nM) for 24 h. Data shown were derived from 2 technique repeats. **B** A schematic illustration of the labeling strategy used for DNA fiber analysis. **C** LIM1215 and Colo205 cells treated with DMSO or IACS-010759 (100 nM) for 24 h were subjected to DNA fiber assays. Data shown are representative microphotographs (lower panels), and mean ± S.D. of the replication fork progression speed of the 100 randomly selected tracts (upper panels) from one representative, of 3 individual experiments, two-tailed Student’s *t* test. Scale bar, 5 μm. **D** Whole cell lysates from LIM1215 and HCT116 cells with or without treatment with IACS-010759 (100 nM) for 24 h were subjected to Western blot analysis. Data shown are representatives of 3 individual experiments. **E** Whole cell lysates from LIM1215 and HCT116 cells transfected with the control siRNA, p53 siRNA1, or p53 siRNA2 were subjected to Western blot analysis. Data shown are representatives of 3 individual experiments. **F**, **G** LIM1215 (upper panels) and HCT116 (lower panels) cells transfected with the control siRNA, p53 siRNA1, or p53 siRNA2 were treated with IACS-010759 (100 nM) followed by cell viability (**F**) or cell cycle progression analysis (**G**). Data shown are mean ± S.D. of results from 3 individual experiments, one-way ANOVA followed by Tukey’s multiple comparison test. **H** LIM1215 (upper panel) and HCT116 (low panel) cells transfected with the control siRNA, p53 siRNA1, or p53 siRNA2 were treated with IACS-010759 (100 nM) followed by DNA fiber assays. Data shown are mean ± S.D. of the replication fork progression speed of the 100 randomly selected tracts (upper panels) from one representative of 3 individual experiments, one-way ANOVA followed by Tukey’s multiple comparison test.

The arrest of cell cycle at S phase following IACS-010759 treatment was strongly suggestive of DNA replication stress (Fig. 1F) [23]. Substantiating this, IACS-010759 treatment enriched the G2/M DNA-damage checkpoint regulation pathway as revealed by ingenuity pathway analysis (IPA) of RNA-sequencing (RNA-seq) data from both LIM1215 and Colo205 cells (Fig. 4A). Moreover, it slowed replication fork progression as revealed in DNA fiber assays (Fig. 4B, C). In accordance, IACS-010759 treatment enriched the p53 signaling pathway in LIM1215 cells (wildtype p53) (Fig. 4A), which is known to play a critical role in the cellular response to replication stress [23, 24]. Indeed, p53 was upregulated, as was its transcription target p21 [25], in LIM1215 and HCT116 cells (wildtype p53) (Fig. 4D). Instructively, siRNA knockdown of p53 attenuated the inhibition of cell viability and reduced the cell cycle arrest at S phase caused by IACS-010759 treatment (Fig. 4E–G). Moreover, it decreased IACS-010759-induced replication fork slowing (Fig. 4H). Therefore, p53 is involved in regulating the response to replication stress in CRC cells when OXPHOS is inhibited.

Noticeably, IACS-010759 treatment-induced moderate increases in the levels of reactive oxygen species (ROS) in both LIM1215 and Colo205 cells (Fig. S3D). Nevertheless, co-treatment with the ROS scavenger N-acetylcysteine (NAC) did not impinge on IACS-010759-induced reductions in the viability of these cells (Fig. S3E), suggesting that ROS do not have a major role in determining the fate of metabolically flexible glycolysis-competent cancer cells upon OXPHOS inhibition.

### Nucleotide deficiencies are involved in inducing replication stress by OXPHOS inhibition

**Fig. 5:**
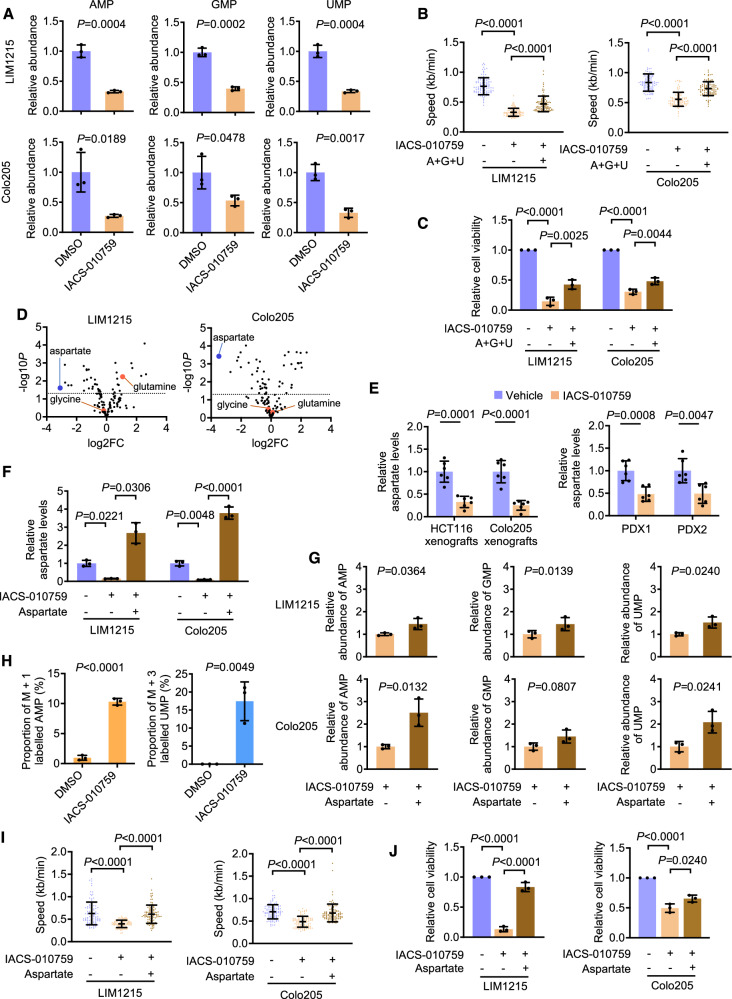
Nucleotide deficiencies are responsible for replication stress caused by OXPHOS inhibition. **A** LIM1215 and Colo205 cells treated with DMSO or IACS-010759 (100 nM) for 24 h were subjected to LC-MS analysis. Data shown are mean ± S.D. of results from 3 technique repeats, two-tailed Student’s *t* test. **B**, **C** LIM1215 and Colo205 cells treated as indicated for 24 h were subjected to DNA fiber assays (**B**), and for 72 h before cell viability assays (**C**). Replication fork progression speed was calculated using 100 randomly selected tracts from one representative of 3 individual experiments. A, adenine; G, guanine; U, uridine. 30 μΜ of each was supplemented. Data shown are mean ± S.D., one-way ANOVA followed by Tukey’s multiple comparison test. **D** Volcano plots of targeted metabolomics data from LIM1215 and Colo205 cells as treated in (**A**). Data shown are average expression levels of metabolites in cells treated with IACS-010759 relative to DMSO, from 3 technique repeats. **E** CRC cells isolated from xenografts in mice treated with vehicle or IACS-010759 were subjected to colorimetric aspartate assays. Data shown are mean ± S.D.; n = 6 mice per group, two-tailed Student’s *t* test. **F**, **G** LIM1215 and Colo205 cells treated with IACS-010759 (100 nM) in the absence or presence of aspartate for 24 h were subjected to targeted metabolomics analysis. Data shown are mean ± S.D. of results from 3 technique repeats, one-way ANOVA followed by Tukey’s multiple comparison test (**F**) and two-tailed Student’s *t* test (**G**). **H** LIM1215 cells cultured in media containing uniformly labeled aspartate were treated with DMSO or IACS-010759 (100 nM) for 24 h before isotope-tracing assays. Data shown are mean ± S.D. of results from 3 technique repeats, two-tailed Student’s *t* test. **I**, **J** LIM1215 and Colo205 cells treated as in (**F**) were subjected to DNA fiber assays (**I**), and cell viability assays after 72 h (**J**). Data shown are mean ± S.D., one-way ANOVA followed by Tukey’s multiple comparison test.

DNA replication requires a sufficient supply of nucleotides, the biosynthesis of which critically relies on metabolites generated with the support of OXPHOS [14, 16]. We therefore tested whether DNA replication stress caused by OXPHOS inhibition in CRC cells is due to a lack of nucleotides. Quantitation of individual nucleotides using LC-MS showed that IACS-010759 treatment resulted in reductions in the purine nucleotides, adenine monophosphate (AMP) and guanine monophosphate (GMP), as well as the pyrimidine nucleotide, uridine monophosphate (UMP), in both LIM1215 and Colo205 cells (Fig. 5A).

To functionally verify the role of nucleotide deficiencies, we supplemented the purine nucleobases adenine and guanine in combination with the pyrimidine nucleoside uridine into the cell culture medium before adding IACS-010759. This treatment reversed, albeit partially, the replication fork slowing and the reductions in cell viability caused by IACS-010759 treatment in both LIM1215 and Colo205 cells (Fig. 5B, C). Together, these results suggested that nucleotide deficiencies are involved in the induction of DNA replication stress and the inhibition of proliferation in CRC cells by targeting OXPHOS.

Since the nucleotide pool is also essential for supporting DNA repair, we tested whether the nucleotide deficiency induced by IACS-010759 treatment also hinders the repair of DNA damage caused by genotoxic insults [26]. Pretreatment with IACS-010759 increased the accumulation of phosphorylated histone H2AX (γH2A.X) foci following treatment with the DNA-damaging drug cisplatin in HCT116 and Colo205 cells (Fig. S3F, G). This indicates that OXPHOS inhibition-induced nucleotide deficiencies indeed impair DNA repair capacity.

We also examined whether OXPHOS inhibition causes mitochondrial DNA (mtDNA) stress by monitoring mtDNA replication and distribution [27, 28]. To test mtDNA replication, we labeled LIM1215 and Colo205 cells, with or without IACS-010759 treatment, using 5-bromo-2′-deoxyuridine (BrdU), followed by immunofluorescence staining with anti-BrdU and anti-Tom20 antibodies [27]. The results showed no significant alterations in mitochondrion-enriched BrdU before and after IACS-010759 treatment (Fig. S4A, B), suggesting that OXPHOS inhibition does not trigger abnormal mtDNA replication in these cells [27]. Supporting this, qPCR analysis of genomic DNA revealed that IACS-010759 treatment did not lead to significant changes in the abundance of four randomly selected mitochondrial genes, including MT-ND3, MT-CYB, MT-CO1, and MT-ATP6 (Fig. S4C). To detect mtDNA distribution, we labeled LIM1215 and Colo205 cells, with or without IACS-010759 treatment, using MitoTracker™ Deep Red and then stained them with SYBR Green I dye [28]. The results showed that IACS-010759 treatment did not cause significant changes in the amount and size of mtDNA nucleotides (Fig. S4D–F). Thus, OXPHOS inhibition does not trigger abnormal mtDNA distribution in these cells. Moreover, treatment with IACS-010759 did not cause noticeable alterations in mitochondrial morphology (Fig. S4G).

### An aspartate shortage contributes to the nucleotide deficiencies upon OXPHOS inhibition

To understand the mechanism through which targeting OXPHOS reduces nucleotide pools, we performed targeted metabolomics using LC-MS. Among the metabolites detected in this analysis that donate carbon and/or nitrogen atoms to nucleobases (Fig. S5A), the levels of aspartate but not glutamine and glycine were decreased in both LIM1215 and Colo205 cells (Figs. 5D and S5B). Kinetic studies using colorimetric assays showed that the reductions in aspartate levels occurred as early as 15 min after exposure to IACS-010759, and by 24 h, the levels were only ~15–25% of those before treatment (Fig. S5C). IACS-010759 treatment also caused marked decreases in aspartate levels in additional CRC cell lines (Fig. S5D), whereas treatment with IM156 similarly decreased aspartate levels in LIM1215 and Colo205 cells (Fig. S5E). CRC cells isolated from HCT116 and Colo205 xenografts as well as PDX1 and PDX2 in mice treated with IACS-010759 exhibited reduced aspartate levels compared to the respective controls (Fig. 5E), consolidating that OXPHOS inhibition blocks aspartate biosynthesis in vivo.

We next examined the functional importance of the reduction in aspartate levels triggered by OXPHOS inhibition. The cellular import of aspartate requires excitatory amino acid transporter 1 (EAAT1) [15, 29]. However, EAAT1 expression is generally low in nonneuronal cells [29], leaving such cells largely unable to take up aspartate at physiological levels [17]. Consistently, EAAT1 expression appeared hardly detectable in CRC cell lines (Fig. S5F). We therefore supplemented the culture medium with aspartate at a supraphysiological concentration of 10 mM, which permits cells with low EAAT1 levels to take up aspartate [15, 17, 30]. LC-MS analysis revealed that aspartate supplementation restored cellular aspartate levels and attenuated the reductions in AMP, GMP, and UMP in LIM1215 cells as well as AMP and UMP in Colo205 cells caused by IACS-010759 treatment (Fig. 5F, G). There was also a trend in the recovery of GMP levels in Colo205 cells (Fig. 5G). Together, these results indicated that the aspartate shortage contributes to the nucleotide deficiency in CRC cells when OXPHOS is inhibited.

To further confirm the role of exogenous aspartate in supporting nucleotide synthesis in CRC cells upon OXPHOS inhibition, we conducted stable isotope-tracing assays with uniformly labeled [^15^N]- or [^13^C]-aspartate at the supraphysiological concentration added to the culture medium of LIM1215 cells (Fig. S5G). This analysis showed that [^15^N] was incorporated into ~10% of AMP, whereas [^13^C] was incorporated into ~15% of UMP in LIM1215 cells after the treatment with IACS-010759 (Fig. 5H), substantiating the replenishment of the nucleotide pool by exogenous aspartate. There was little incorporation of [^15^N]- and [^13^C] into nucleotides in cells without IACS-010759 treatment (Fig. 5H), suggesting that de novo aspartate synthesis is sufficient for generating nucleotides in cells with intact OXPHOS.

The supplementation of exogenous aspartate abolished replication fork slowing caused by treatment with IACS-010759 in LIM1215 and Colo205 cells (Fig. 5I), suggesting that the levels of nucleotides had reached the thresholds necessary for replication fork progression. Nevertheless, while it almost completely rescued LIM1215 cells from IACS-010759 treatment-induced proliferation inhibition, its effect on the inhibition of proliferation was less prominent in Colo205 cells (Fig. 5J). Therefore, while the aspartate shortage is an important determinant of replication stress caused by targeting OXPHOS in glycolysis-competent CRC cells, other mechanisms are also likely involved in the inhibition of proliferation in CRC cells upon OXPHOS inhibition.

As both p53 knockdown and aspartate supplementation reduced the inhibition of cell viability and lessened the decrease in replication fork progression caused by IACS-010759 treatment (Figs. 4F, H and 5I, J), it is likely that p53 affects aspartate levels in CRC cells. Alternatively, the addition of exogenous aspartate might influence p53 signaling. However, we did not observe any significant changes in aspartate levels in LIM1215 and HCT116 cells following siRNA knockdown of p53 (Fig. S5H). On the other hand, aspartate supplementation alone did not cause significant changes in the expression of p53 and its transcription target, p21 (Fig. S5I).

### Targeting GOT1 sensitizes glycolysis-competent CRC to OXPHOS inhibition

**Fig. 6:**
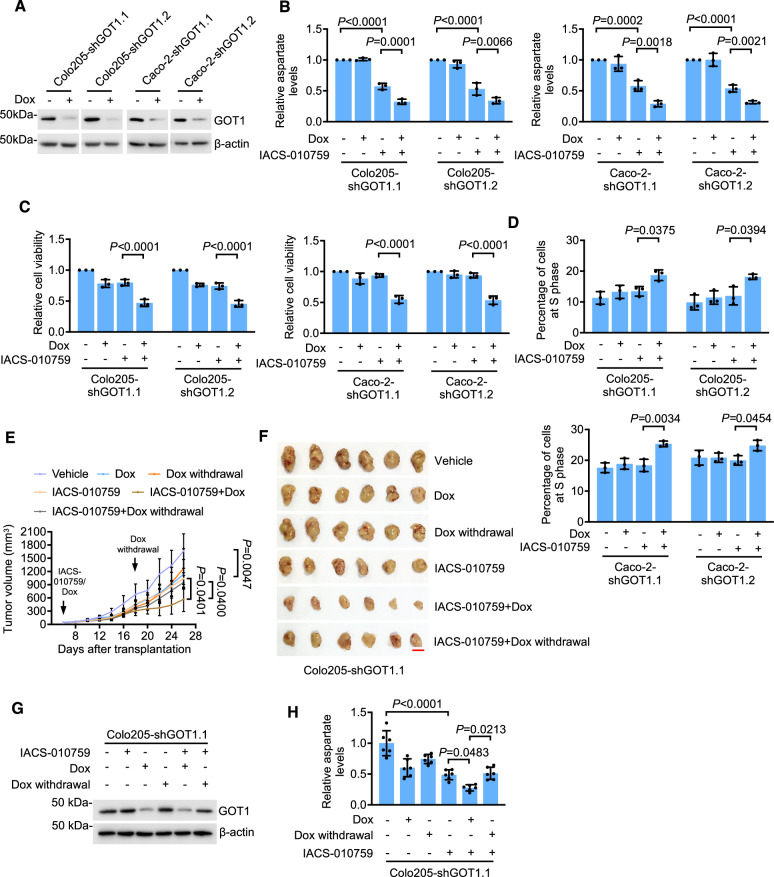
Targeting GOT1 sensitizes glycolysis-competent CRC cells to OXPHOS inhibition. **A** Whole cell lysates from Colo205 and Caco-2 cells transduced with inducible GOT1 shRNA with or without treatment with Dox (200 ng/ml) for 48 h were subjected to Western blot analysis. Data shown are representatives of three individual Western blotting experiments. **B** Colo205 and Caco-2 cells, carrying inducible GOT1 shRNA with or without treatment with Dox for 48 h followed by IACS-010759 treatment (100 nM; 2 h), were subject to colorimetric aspartate assays. Data shown are mean ± S.D. of 3 individual experiments, one-way ANOVA followed by Tukey’s multiple comparison test. **C**, **D** Colo205 and Caco-2 cells carrying inducible GOT1 shRNA with or without treatment with Dox in combination with IACS-010759 treatment (10 nM) for 72 h were subjected to cell viability assays (**C**) and cell cycle progression analysis (**D**). Data shown are mean ± S.D. of results from 3 individual experiments, one-way ANOVA followed by Tukey’s multiple comparison test. **E**, **F** Growth curves (**E**) and photographs (**F**) of xenografts of Colo205 cells carrying inducible GOT1 shRNA1 in NOD/SCID mice treated with the vehicle or IACS-010759 (10 mg/kg) with or without co-treatment with Dox (2 mg/ml supplemented with 10 mg/ml sucrose in drinking water) and the cessation of Dox treatment. Data shown are mean ± S.D. of results from 6 mice per group, one-way ANOVA followed by Tukey’s multiple comparison test. Scale bar: 1 cm. **G** Whole cell lysates of CRC cells isolated from Colo205 xenografts carrying inducible GOT1 shRNA1 in NOD/SCID mice treated as in (**E**) were subjected to Western blot analysis. Data shown are representatives of results from 6 mice per group. **H** CRC cells isolated from Colo205 xenografts carrying inducible GOT1 shRNA1 in NOD/SCID mice treated as in (**E**) were subjected to colorimetric aspartate assays. Data shown are mean ± S.D. of results from 6 mice per group, one-way ANOVA followed by Tukey’s multiple comparison test.

When mitochondrial biosynthesis of aspartate is blocked, GOT1 catalyzes cytosolic production of aspartate (Fig. S6A) [14]. We therefore reasoned that GOT1 plays a role in protecting against OXPHOS inhibition through maintaining aspartate levels. To test this, we established the Colo205 and Caco-2 sub-lines, Colo205-shGOT1 and Caco-2-shGOT1, respectively, with GOT1 conditionally knocked down in response to Dox (Fig. 6A). Inducing GOT1 knockdown with Dox further reduced aspartate levels when treated with a relatively low concentration of IACS-010759 (10 nM) (Fig. 6B). Moreover, it rendered Colo205-shGOT1 and Caco-2-shGOT1 cells more susceptible to IACS-010759-induced inhibition of proliferation, accompanied with increased cell cycle arrest at S phase (Fig. 6C, D).

In an in vivo test, NOD/SCID mice carrying Colo205-shGOT1.1 xenografts were treated with Dox, IACS-010759 (10 mg/kg), or a combination of Dox and IACS-010759. The combined treatment effectively inhibited Colo205-shGOT1 xenograft growth compared to individual treatments (Figs. 6E–G and S6B), associated with a further decrease in the proportion of Ki67-positive cells (Fig. S6C, D). Moreover, CRC cells isolated from mice co-treated with Dox and IACS-010759 exhibited a greater reduction in aspartate levels (Fig. 6H). Cessation of Dox treatment led to the recovery of GOT1 expression and restored tumor growth (Figs. 6E–G and S6B), indicating that the growth changes were linked to the levels of GOT1. Thus, targeting GOT1 is a potential approach to sensitize glycolysis-competent CRC to OXPHOS inhibitors.

## Discussion

Despite the recently demonstrated dose-limiting toxicities of several mitochondrial complex I inhibitors in patients [7], the potential of targeting OXPHOS for cancer treatment remains explorable through approaches such as combinations to achieve therapeutic benefits at doses attainable in clinical settings and selectively inhibiting OXPHOS in cancer cells [31]. However, CRC cells often exhibit the glycolytic phenotype [11], making them ostensibly less vulnerable to OXPHOS inhibition. Nevertheless, our results in this study revealed that glycolytically competent CRC cells are susceptible to targeting OXPHOS in vitro and in preclinical PDX CRC models. Moreover, we show that GOT1 plays an important role in protecting CRC cells against OXPHOS inhibition, proposing GOT1 targeting as a potential strategy to improve the therapeutic efficacy of OXPHOS inhibitors in cancer treatment.

Targeting OXPHOS did not significantly reduce cellular ATP levels in glycolysis-competent CRC cells as it did in glycolytic-deficient cancer cells. This outcome was clearly due to the metabolic switch towards glycolysis when OXPHOS was inhibited. What, then, is the mechanism responsible for diminishing proliferation of these cells when OXPHOS is inhibited? Our results pinpointed the role of DNA replication stress, evidenced by cell cycle arrest at S phase, replication fork slowing, and activation of the p53 pathway. Furthermore, we discovered that this replication stress was caused by nucleotide deficiencies resulting from a shortage of aspartate. This finding is consistent with previous results showing that enabling aspartate biosynthesis is an essential function of the mitochondrial electron transport chain in proliferating cells [14, 16]. Moreover, treatment with IACS-010759 reduced aspartate production, impairing nucleotide biosynthesis in AML cells [3].

As the “guardian of the genome”, p53 is known to function to arrest cell cycle progression, allowing sufficient time for the replication fork slowing to be resolved, or, in the case of unresolvable inhibition of replication fork progression, eliminating cells through inducing apoptosis [24]. Our results align with this well-established role of p53 in response to replication stress by showing that glycolysis-competent CRC cells harboring wildtype p53 were more sensitive to IACS-010759 than those carrying mutant p53 in short-term cell viability assays. Nevertheless, this difference diminished in clonogenic experiments, indicating that mechanisms other than p53 signaling play important roles in determining the ultimate fate of CRC cells upon OXPHOS inhibition. Regardless, p53 knockdown mitigated IACS-010759-induced replication fork stalling and inhibition of proliferation, consistent with the notion that cellular response to replication stress is impaired when p53 is defective [32]. As a tumor suppressor, p53 loss-of-function mutations occur in ~50% of CRCs [33]. Additionally, the expression of wildtype p53 is frequently suppressed at the posttranslational level by mechanisms such as the high expression of the E3 ubiquitin ligase human double minute 2, and the long noncoding RNA MILIP in CRCs [19, 34]. Restoring p53 function using small molecules such as APR-246 and Nutlin-3a may represent a potential approach for sensitizing CRCs to OXPHOS targeting [33, 34].

It is intriguing to note that inhibition of OXPHOS triggered replication fork slowing to a greater extent in wildtype compared to mutant p53 CRC cells. This observation raises the possibility that p53 per se also contributes to the decreases in aspartate levels and the induction of replication stress in CRC cells when OXPHOS is inhibited. P53 can regulate aspartate biosynthesis indirectly through promoting the generation of metabolic intermediates to fuel the tricarboxylic acid cycle (TCA) cycle through mechanisms such as transcriptional activation of glutaminase 2, an enzyme that catalyzes the conversion of glutamine to glutamate [35]. The latter is then converted to α-ketoglutarate, which enters the TCA cycle and ultimately contributes to the production of oxaloacetate for the generation of aspartate through transamination [14]. Indeed, p53^-/-^ HCT116 cells exhibited reduced aspartate levels compared with the p53^+/+^ counterparts [36]. However, we did not observe any significant changes in aspartate levels in CRC cells when p53 was knocked down by siRNA. This discrepancy suggests that the effect of p53 on aspartate levels is conceivably context dependent. In accordance, our results showed no significant differences in the extents of reductions in aspartate levels between wildtype and mutant p53 CRC cells following IACS-010759 treatment, suggesting that targeting OXPHOS inhibits aspartate biosynthesis in CRC cells irrespective of their p53 statuses. Supporting this, albeit indirectly, is the observation that glutamine consumption was comparably reduced in both wildtype and mutant p53 CRC cells when OXPHOS was inhibited. Collectively, our results support the notion that the lack of aspartate triggers replication stress, while p53 regulates the response to this stress in CRC cells when OXPHOS is inhibited.

Although our results have established the important role of an aspartate shortage in the induction of nucleotide deficiencies, leading to DNA replication stress and subsequently inhibition of proliferation in CRC cells when OXPHOS is inhibited, other mechanisms, such as the blockage of de novo lipid biosynthesis, may also be involved as shown in previous studies [37]. Furthermore, as a nucleotide precursor, aspartate is similarly required for mtDNA biogenesis [16]. However, we did not detect any significant changes in mtDNA replication and distribution in CRC cells after treatment with IACS-010759, suggesting that OXPHOS inhibition does not cause mtDNA stress that otherwise conceivably contributes to inhibition of proliferation [38, 39]. Similarly, our results showed that IACS-010759 treatment did not induce significant changes in mitochondrial morphology, indicating that OXPHOS inhibition does not alter mitochondrial dynamics in CRC cells. It has been previously shown that IACS-010759 treatment promoted the elongation of mitochondria, implicating increased mitochondrial fusion, in a cell line-dependent fashion [40].

An important finding of this study that is potentially relevant to practice is that inhibiting cytosolic aspartate biosynthesis by targeting GOT1 is likely an approach to sensitize glycolysis-competent CRC to OXPHOS inhibitors. Indeed, a previous study using CRISPR-based genetic screening identified the expression of GOT1 as an important mechanism of aspartate production when OXPHOS is inhibited [14]. The combination of targeting of GOT1 and IACS-010759 reduced its concentration required to effectively inhibit CRC growth, suggesting that this combination may mitigate the dose-limiting toxicities of OXPHOS inhibitors. With the development of GOT1 specific inhibitors [41], it is anticipated that further investigations of the combination of specific GOT1 and OXPHOS inhibitors in preclinical models would be conducted in the near future.

## Supplementary information

Supplementary Information

Uncropped Western Blots

## Data Availability

The data generated during the current study are available in Gene Expression Omnibus (GEO) at GSE244319 and in Metabolomics Workbench at DataTrack ID 4477. All other underlying data for the manuscript are available from the corresponding author XDZ or LJ upon reasonable request.
