# Supplementary material for: DNA replication stress underpins the vulnerability to oxidative phosphorylation inhibition in colorectal cancer
Source: Cell Death Dis. 2025 Jan 14;16(1):16. doi: 10.1038/s41419-025-07334-4 (PMC11733219; doi:10.1038/s41419-025-07334-4)
Supplement: Supplementary file 1 — Supplementary Information [file 41419_2025_7334_MOESM1_ESM.pdf]

Supplementary Information for

DNA replication stress underpins the vulnerability to oxidative phosphorylation inhibition in colorectal cancer

Xiao Hong Zhao,[1] Man Man Han,[2,3] Qian Qian Yan,[2,3] Yi Meng Yue,[2,3] Kaihong Ye, [1,3] Yuan Yuan Zhang,[1] Liu Teng,[1,2,3] Liang Xu,[1] Xiao-Jing Shi,[2] Ting La,[1,4] Yu Chen Feng,[5] Ran Xu,[5] Vinod K Narayana,[6,7] David P De Souza,[6,7] Lake-Ee Quek,[8] Jeff Holst,[9] Tao Liu,[2,3,10] Mark A. Baker,[1] Rick F. Thorne,[1,2,3] Xu Dong Zhang,[1,2,3]* Lei Jin[2,3,5]*

Xu Dong Zhang or Lei Jin

Email: Xu.Zhang@newcastle.edu.au or Lei.Jin@newcastle.edu.au

This PDF file includes:

Supplementary text

Figures S1 to S6

Tables S1 to S5

Supplementary Information Text

Materials and Methods

Cell cycle

Cell cycle analysis was conducted using PI staining, as previously described [1]. In brief, cells were fixed by 70% ethanol on ice for 2 hours and then centrifuged at 1500 × g. Cell pellets were re-suspended in PBS containing 0.1% NaN_3, 1% FBS and then incubated on ice for 15 minutes. After discarding the supernatant, the cell pellet was resuspended in 0.5 ml of PBS containing 10 μg/ml RNase A and 20 μg/ml PI working solution, and subsequently incubated at room temperature in the dark for 30 minutes. Cells were then subjected to analysis using a flow cytometer (FACSCanto, BD Biosciences).

Colony formation

500-2000 cells were seeded and incubated in a 6-well plate. Around two weeks later, cells were fixed, stained with crystal violet, and photographed [2, 3].

Plasmids

The FH1-tUTG plasmid was a kind gift from Professor Herold MJ (Walter and Eliza Hall Institute of Medical Research, Australia). The pMDLg/pRRE plasmid (#12251), pMD2.g plasmid (#12259) and pRSV-Rev plasmid (#12253) were purchased from Addgene (Watertown, MA). The pcDNA3.1(+) plasmid (#V79020) was purchased from Invitrogen (Waltham, MA). The pcDNA3.1(+)- GOT1 plasmid was constructed by Genewiz (Burlington, MA). The pcDNA3.1-Flag-p53 plasmid was kindly provided by Professor Mian Wu and A/Prof. Wanglai Hu (Translational Research Institute, Henan Provincial People's Hospital, China).

RNA sequencing

RNA-seq was conducted by Novogen (Singapore). RNA libraries for RNA-seq were prepared using NEBNext® Ultra™ RNA Library Prep Kit (#E7770; New England Biolabs, Ipswich, MA). In brief, mRNA was isolated from total RNA (0.4 mg) using magnetic beads with attached poly-T oligos. After fragmentation, the first strand cDNA was synthesized using random hexamer primers, followed by the second strand cDNA synthesis using deoxyuridine triphosphate for directional library. The quality of the library was monitored using the Qubit RNA Integrity and Quality Assay kit (#Q33221; ThermoFisher) and qPCR, whereas size distribution detection was done using the Agilent 2100 Bioanalyzer (Agilent). Libraries were then pooled and sequenced on the Illumina NovaSeq 6000 System (Illumina, San Diego, CA). Index of the reference genome was built using Hisat2 v2.0.5 and paired-end clean reads were aligned to the reference genome. FeatureCounts v1.5.0-p3 was used to count the reads numbers mapped to each gene. Fragments Per Kilobase of transcript per Million mapped reads of each gene was then calculated based on the length of the gene and reads count mapped to the gene. Differential expression analysis of two conditions/groups (two biological replicates per condition) was performed using the DESeq2 R package v1.20.0. Genes with adjusted *P*-value less than 0.05 were marked as differentially expressed.

Extracellular acidification rate (ECAR) and oxygen consumption rate (OCR) measurements

ECAR and OCR assays were performed with the Seahorse XF Glycolysis Stress Test Kit (#103020-100) and Seahorse XF Cell Mito Stress Test Kit (#103015-100), respectively, using a Seahorse XF96 analyzer (Agilent, Santa Clara, CA) according to the

manufacturer's instructions. Briefly, 6000 cells/well were seeded in a 96-well XF cell culture microplate in culture medium 24 hours before assay. ECAR was measured with an XF96 analyzer in XF base medium (pH 7.4) containing 2 mM glutamine following sequential additions of glucose (10 mM), oligomycin (1 μM) and 2-DG (50 mM), and OCR, in XF base medium (pH 7.4) containing 2 mM glutamine and 10 mM glucose following sequential additions of oligomycin (1.5 μM), FCCP (1 μM), and Rot+AA (0.5 μM). Data were analysed using the Seahorse XF Glycolysis Stress Test Report Generator package or XF Cell Mito Stress Test Report Generator package with results normalised against cell number determined using a Countstar® BioTech Automated Cell Counter (Alit Biotech, Shanghai, China).

Cellular ATP, lactate, aspartate, ROS level measurements

ATP, lactate, aspartate and ROS levels were measured with Luminescent ATP detection assay (#ab113849, Abcam), Lactate-Glo™ assay (#J5021, Promega), and Aspartate assay (#ab102512, Abcam) kits, respectively, using a BioTek Synergy 2 Multidetection Microplate Reader (Agilent, Santa Clara, CA), according to the manufacturer's instructions. ROS levels were also measured with a ROS assay kit (#C10422, Life Technologies, Carlsbad, CA) using a flow cytometer (FACSCanto, BD Biosciences).

Glucose and glutamine consumption and extracellular lactate assays

Extracellular glucose, glutamine, or lactate levels were measured with colorimetric Glucose assay (#GAGO20, Sigma-Aldrich), Glutamine assay (#ab197011, Abcam), and Lactate assay (#MAK064, Sigma-Aldrich) kits, respectively, using a BioTek Synergy 2 Multidetection Microplate Reader (Agilent, Santa Clara, CA), according to the manufacturer's instructions.

Mitochondrial complex I and II activity

Mitochondrial complex I and II activity were measured with the MitoCheck® Complex I Activity Assay kit (#700930, Cayman Chemical) and the Complex II Enzyme Activity Microplate Assay kit (#ab109908, Abcam), respectively, according to the manufacturers' instructions using a BioTek Synergy 2 Multidetection Microplate Reader (Agilent, Santa Clara, CA).

DNA fiber assays

DNA fiber assays were carried out as previously described [4]. Cells were first labeled with 25 μM 5-Iodo-2'-deoxyuridine (IdU) for 10 minutes, then with 250 μM 5-chloro-2′-deoxyuridine (CldU) for 20 minutes at 37°C. After removal of the medium, cells were washed, trypsinized, and suspended in cold PBS. Cells were applied onto slides, lysed, air-dried, and fixed. DNA fibers were denatured, blocked, and labeled with specific antibodies for IdU and CIdU tracks. Slides were washed, dried, mounted, and observed under a Zeiss AXio fluorescence microscope (Macquarie Park, NSW, Australia) using ×100 oil-immersion lenses, capturing 100 fibers per experimental condition. The length of DNA fibers under a particular condition was recorded as the average of the lengths of the 100 fibers measured using ImageJ software (National Institute of Health). The fork progression speed was calculated as 1 μm = 2 kb as described previously [4].

Western blotting

The procedure has been described previously [5, 6]. In brief, cells were harvested and lysed with cell lysis buffer [10mM Tris-HCl (pH7.6), 140mM NaCl, 0.5mM $CaCl_2$, 0.5mM $MgCl_2$, 0.02% NaN_3, 1% Triton X-100 and a proteinase inhibitor cocktail (#12352200,

Roche)]. Equal amounts of the protein extracts were subjected to SDS-PAGE and analyzed by Western blotting.

Immunohistochemistry (IHC)

IHC was carried out as previously described [5]. The percentage of positive cells was estimated on an arbitrary scale of 0-4: no positive cells (0), <10% of positive cells (1), 10-50% positive cells (2), 51-80% positive cells (3), and > 80% positive cells (4). The intensity of staining (intensity score) was judged on an arbitrary scale of 0 to 3: no staining (0), weakly positive staining (1), moderately positive staining (2), strongly positive staining (3). An immunoreactive score (IRS) was derived by multiplying the score of percentage of positive cells with staining intensity. Researchers were blinded to the group allocation when quantitating immunostaining.

Targeted metabolomics

Targeted metabolomics was carried out using liquid chromatography-mass spectrometry (LC-MS) analysis. Cells were washed with milli-Q water at 37°C, then snap-frozen by covering the plate in liquid nitrogen. Polar metabolites were extracted using chloroform: methanol (1:9, v/v, 500 µl) containing internal standards ($^{13}C_6$-Sorbitol, $^{13}C_5$, ^{15}N-Valine and $^{13}C_6$-Leucine) at 4 µM per sample. After incubation at 4°C on a thermomixer (950 rpm) for 10 min followed by centrifugation at 14000 rpm at 4°C for 5 min, the supernatant was transferred into high-performance liquid chromatography (HPLC) glass vials with inserts for LC-MS analysis. Polar metabolite analysis was conducted using polar hydrophilic interaction liquid chromatography (pHILIC)-LC-MS on the Agilent 1200 series LC system (Agilent) and the mass spectrometry analysis was performed on a 6545B series quadrupole time-of-flight mass spectrometer (QTOF MS; Agilent) as described previously [7, 8]. Peak

area integration and targeted data matrix were generated on the retention time and molecular masses matching to the authentic standards (Metabolomics Standard Initiative) level 1 for each metabolite using MassHunter TOF Quantitative Analysis Software (Version V.09.00; Agilent) [9].

Stable isotope tracing

The culture medium of cells seeded onto 6-well plates with or without treatment as desired was supplemented with uniformly labeled [^{15}N]- or uniformly-labelled [$^{13}C_4$]-aspartate (10 mM). After incubation for the indicated period, cells were washed with ice-cold PBS and snap frozen with liquid nitrogen. Metabolites were extracted using the acidic acetonitrile-methanol method as described before [10]. These extracts were then analyzed by LC-MS using a Q Exactive HF-X quadrupole-orbitrap mass spectrometer on a Vanquish UHPLC system (Thermo Scientific), with chromatographic separation achieved on an Poroshell 120 HILIC-Z column (2.1×150 mm, 2.7 μm, Agilent). Buffer A was 20 mM ammonium acetate, 20 mM ammonium hydroxide and 5% acetonitrile; Buffer B was 100% acetonitrile and the run time was 30 min with a flowrate of 200 μL/min. Mass isotopologues of amino acids and nucleotides were extracted using MSConvert and MATLAB scripts as previously described [11]. Detected mass shifts were verified by checking against unlabeled cell extracts and metabolite standards.

Mouse models

For LIM1215 and Colo205 xenograft models, cells were subcutaneously injected into the right flanks of 6-week-old female NOD/SCID gamma (NSG) mice (Australian BioResources, Australia). On day 6 after transplantation when tumors became palpable, mice carrying tumors derived from each cell line were randomized into two groups, using

function Rand() in Excel, with 6 mice in each group that were subjected respectively to treatments with the vehicle control (0.5% methyl cellulose in 200 μl sterile deionized water) or IACS-010759 (15 mg/kg in 200 μl 0.5% methyl cellulose; three days on, one day off) via oral gavage. In the case of xenograft models with Colo205 cells expressing an inducible GOT1 shRNA system (Colo205-shGOT1.1) in response to Dox, cells were subcutaneously injected into the right flanks of 7-week-old male NOD/SCID mice (Gempharmatech, Nanjing, China). On day 6 after transplantation when tumors became palpable, mice were randomized into six groups with 6 mice in each group that were subjected respectively to treatments with the vehicle control (0.5% methyl cellulose in 200 μl sterile deionized water; oral gavage, three days on, one day off), Dox (2 mg/ml in drinking water), Dox for 12 days followed by withdrawal of Dox, IACS-010759 (10 mg/kg in 200 μl 0.5% methyl cellulose; oral gavage, three days on, one day off), Dox plus IACS-010759, and Dox plus IACS-010759 with withdrawal of Dox 12 days after the first treatment. Mice were sacrificed on day 26 after transplantation in these xenograft experiments. To establish CRC PDX models, tumor tissue blocks (3 mm × 3 mm) were transplanted subcutaneously into the right dorsal flanks of 7-week-old male NOD/SCID mice (Gempharmatech, Nanjing, China). On day 10 after transplantation, mice were subjected to treatments with the vehicle control (0.5% methyl cellulose in 200 μl sterile deionized water) or IACS-010759 (15 mg/kg in 200 μl 0.5% methyl cellulose; three days on, one day off) via oral gavage. Mice were sacrificed on day 60 after transplantation. Tumor growth was measured every 2 days using a caliper. Mice were euthanized through CO_2 inhalation followed by cervical dislocation. In some experiments, pimonidazole (60 mg/kg in saline) was injected via i.p. three hours before euthanasia. Tumors were then excised and weighed, with each tumor divided into two pieces. One piece was processed for FFPE tumor blocks, and another was used for isolation of human CRC cells. The

Animal Research Ethics Committee permits a maximal tumor size of 2000 mm^3. No tumors exceeded this limit in any of the relevant experiments.

Purification of human CRC cells from xenografts

Purification of human CRC cells from tumor xenografts was conducted following a previously described protocol [12]. In brief, tumor tissues harvested from mice were immediately transferred in ice-cold Hanks' Balanced Salt solution (HBSS; #H8264, Sigma-Aldrich). The tissues were cut, minced, and incubated in 0.1% trypsin solution on a shaker at 4°C for 20 hours. The tissues were then transferred into DMEM medium and incubated in a warm water bath at 37°C for 15 minutes. This was followed by incubation in HBSS containing 0.1% collagenase solution (#9001-12, Sigma-Aldrich) at 37°C for 45 minutes. The resulting tissue fragments were collected and filtered. Single cells were harvested through centrifugation. CRC cells were purified using FACS (BD FACS Air II) with a labeled antibody against human EpCAM.

Immunofluorescence (IF)

Cells grown on coverslips were washed three times with PBS and then fixed with 4% paraformaldehyde for 15 min at room temperature. Following fixation, the cells were washed with PBS and permeabilized for 15 min using permeabilization buffer (0.2% Triton X-100 in PBS containing 10% BSA). For the 5-bromo-2'-deoxyuridine incorporation assay, the cells were incubated with 50 μM BrdU for 16 hours [13], then fixed, permeabilized and treated with HCl 2 N for 20 min at 37 °C. Cells were then blocked with PBS containing 5% FBS for 1 hour at room temperature before incubation overnight at 4°C with primary antibody. After washing three times with PBS, cells were incubated with

secondary antibody for 2 hours in the dark. After washing with PBS, the coverslip was mounted and visualized by confocal microscope.

Analysis of mitochondrial DNA

Cells were plated onto Cellview^{TM} cell culture dishes (Greiner) and treated with either DMSO or IACS-010759 for 24 hours. Following treatment, cells were stained with MitoTracker Deep Red according to manufacturer's protocol. For mtDNA staining, SYBR Green I was diluted 1:100,000 in pre-warmed cell culture medium, and the cells were incubated with SYBR Green I solution at 37 °C for 15 min in a CO_2 incubator [14]. After incubation, the cells were washed three times with cell culture medium. Live cells were visualized by confocal microscope (LSM900, Zeiss) with a 63× oil objective.

Quantitative PCR (qPCR)

Genomic DNA isolated using a PureLink^{TM} Genomic DNA Mini Kit (Invitrogen) was subjected to PCR analysis. The $2^{-\Delta\Delta CT}$ method was used to calculate the relative gene expression levels in comparison to the RPL13A housekeeping control. Primer sequences are listed in Table S5.

References

1 La T, Chen S, Zhao XH, Zhou S, Xu R, Teng L *et al*. LncRNA LIMp27 Regulates the DNA Damage Response through p27 in p53-Defective Cancer Cells. *Adv Sci (Weinh)* 2023; 10: e2204599.

2 Feng YC, Liu XY, Teng L, Ji Q, Wu Y, Li JM *et al*. c-Myc inactivation of p53 through the pan-cancer lncRNA MILIP drives cancer pathogenesis. *Nat Commun* 2020; 11: 4980.

3 Wen Mi JY, Liucheng Li, Lingzhi Zhu, Xinyi Xia, Li Yang, Fei Li, Yi Xu, Junfeng Bi, Pingyu Liu, Li Chen, Fuming Li. BET inhibition induces GDH1-dependent glutamine metabolic remodeling and vulnerability in liver cancer. *Life Metabolism* 2024; 3.

4 Nakatani T, Lin J, Ji F, Ettinger A, Pontabry J, Tokoro M *et al*. DNA replication fork speed underlies cell fate changes and promotes reprogramming. *Nat Genet* 2022; 54: 318-327.

5 Wang PL, Teng L, Feng YC, Yue YM, Han MM, Yan Q *et al*. The N-Myc-responsive lncRNA MILIP promotes DNA double-strand break repair through non-homologous end joining. *Proc Natl Acad Sci U S A* 2022; 119: e2208904119.

6 Huan Chen TL, Di Chen, Wenjuan Liu, Huan Qi, Tian Xia, Xiaolong Liu, Wen Wang, Xin Guo, Wuxiyar Otkur, Fangjun Wang, Zhaochao Xu, Jean-Claude Martinou, Hai-long Piao. Mitochondrial YBX1 promotes cancer cell metastasis by inhibiting pyruvate uptake. *Life Metabolism* 2023; 2.

7 Kong G, Ellul S, Narayana VK, Kanojia K, Ha HTT, Li S *et al*. An integrated metagenomics and metabolomics approach implicates the microbiota-gut-brain axis in the pathogenesis of Huntington's disease. *Neurobiol Dis* 2021; 148: 105199.

8 Jang C, Chen L, Rabinowitz JD. Metabolomics and Isotope Tracing. *Cell* 2018; 173: 822-837.

9 Sumner LW, Amberg A, Barrett D, Beale MH, Beger R, Daykin CA *et al*. Proposed minimum reporting standards for chemical analysis Chemical Analysis Working Group (CAWG) Metabolomics Standards Initiative (MSI). *Metabolomics* 2007; 3: 211-221.

10 Lu W, Wang L, Chen L, Hui S, Rabinowitz JD. Extraction and Quantitation of Nicotinamide Adenine Dinucleotide Redox Cofactors. *Antioxid Redox Signal* 2018; 28: 167-179.

11 Quek LE, van Geldermalsen M, Guan YF, Wahi K, Mayoh C, Balaban S *et al*. Glutamine addiction promotes glucose oxidation in triple-negative breast cancer. *Oncogene* 2022; 41: 4066-4078.

12 Ali MY, Anand SV, Tangella K, Ramkumar D, Saif TA. Isolation of Primary Human Colon Tumor Cells from Surgical Tissues and Culturing Them Directly on Soft Elastic Substrates for Traction Cytometry. *J Vis Exp* 2015: e52532.

13 Pantic B, Ives D, Mennuni M, Perez-Rodriguez D, Fernandez-Pelayo U, Lopez de Arbina A *et al*. 2-Deoxy-D-glucose couples mitochondrial DNA replication with mitochondrial fitness and promotes the selection of wild-type over mutant mitochondrial DNA. *Nat Commun* 2021; 12: 6997.

14 Sasaki T, Sato Y, Higashiyama T, Sasaki N. Live imaging reveals the dynamics and regulation of mitochondrial nucleoids during the cell cycle in Fucci2-HeLa cells. *Sci Rep* 2017; 7: 11257.

Fig. S1

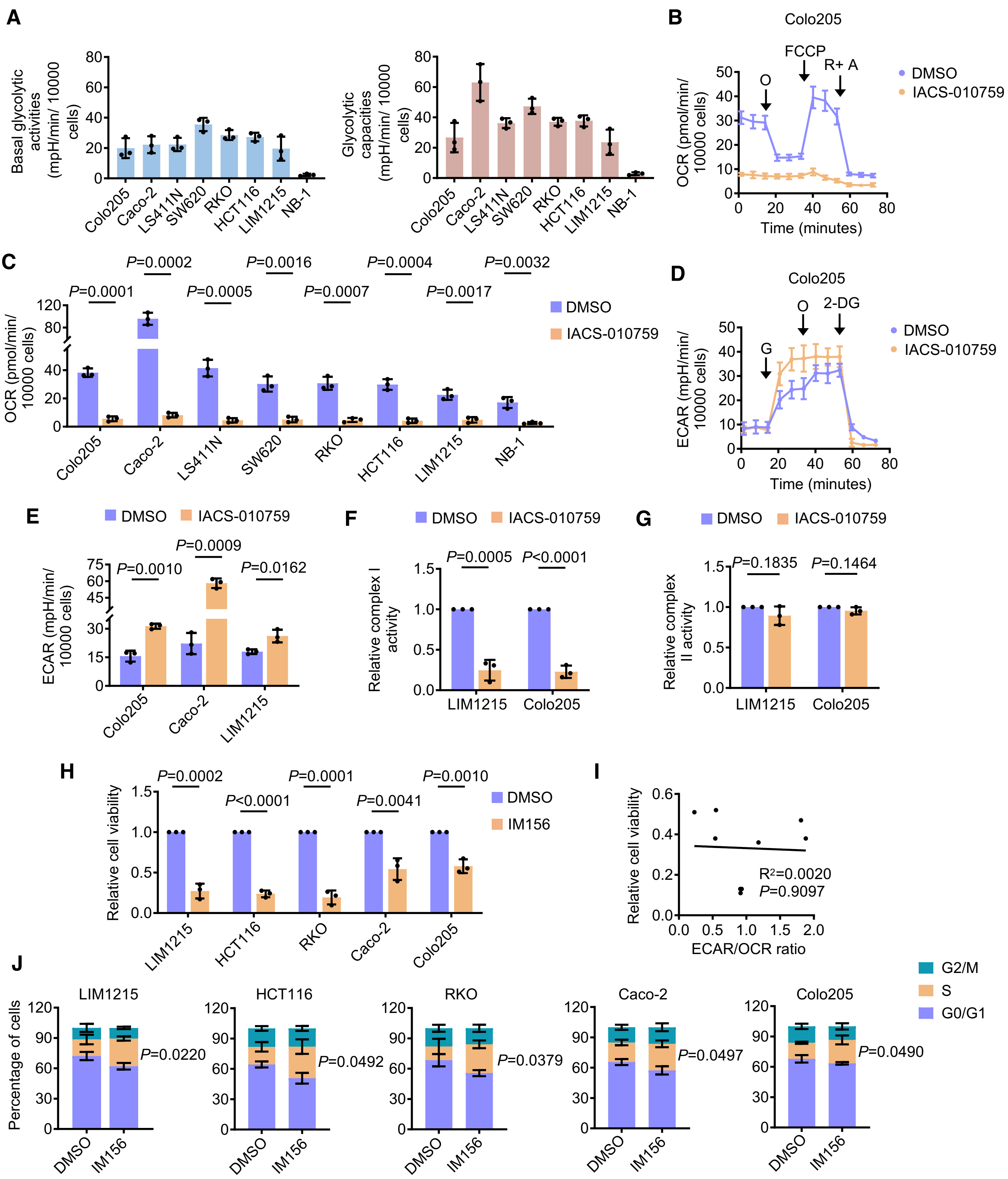

A
Basal glycolytic activities (mpH/min/ 10000 cells)
Glycolytic capacities (mpH/min/ 10000 cells)
Colo205
Caco-2
LS411N
SW620
RKO
HCT116
LIM1215
NB-1
B
Colo205
OCR (pmol/min/ 10000 cells)
O
FCCP
R+ A
DMSO
IACS-010759
Time (minutes)
C
P=0.0001
P=0.0002
P=0.0005
P=0.0016
P=0.0007
P=0.0004
P=0.0017
P=0.0032
OCR (pmol/min/ 10000 cells)
D
Colo205
ECAR (mpH/min/ 10000 cells)
G
O
2-DG
Time (minutes)
E
P=0.0010
P=0.0009
P=0.0162
ECAR (mpH/min/ 10000 cells)
F
P=0.0005
P<0.0001
Relative complex I activity
G
P=0.1835
P=0.1464
Relative complex II activity
H
P=0.0002
P<0.0001
P=0.0001
P=0.0041
P=0.0010
Relative cell viability
IM156
I
Relative cell viability
R2=0.0020
P=0.9097
ECAR/OCR ratio
J
Percentage of cells
P=0.0220
P=0.0492
P=0.0379
P=0.0497
P=0.0490
G2/M
S
G0/G1

Fig. S1. OXPHOS inhibition induces S phase cell cycle arrest in glycolysis-competent CRC cells

(**A**) The basal glycolytic activity and the glycolytic capacity of each of the indicated CRC cell lines and the NB-1 neuroblastoma cell line were measured. Data shown are mean ± S.D. of results from 3 individual experiments.

(B) Colo205 cells treated with the DMSO or IACS-010759 (100 nM) for 6 hours were subjected to measurement of the OCR. Data shown are representatives of 3 individual experiments.

(C) CRC cells and NB-1 neuroblastoma cells treated as in **B** were subjected to measurement of the OCR. Data shown are mean ± S.D. of results from 3 individual experiments, two-tailed Student's *t* test.

(D) Colo205 cells treated as in **B** were subjected to measurement of the ECAR. Data shown are representatives of 3 individual experiments.

(E) CRC cells treated with as in **B** were subjected to measurement of the ECAR. Data shown are mean ± S.D. of results from 3 individual experiments, two-tailed Student's *t* test.

(F and G) LIM1215 and Colo205 cells treated with DMSO or IACS-010759 (100 nM) for 24 hours were subjected to colorimetric complex I (**F**) and complex II (**G**) activity assays. Data shown are mean ± S.D. of results from 3 individual experiments, two-tailed Student's *t* test.

(H) CRC cells treated with DMSO or IM156 (15 μM) for 72 hours were subject to cell viability assays. Data shown are mean ± S.D. of results from 3 individual experiments, two-tailed Student's *t* test.

(I) Regression analysis of the relationship between the sensitivity of CRC cell lines to IACS-010759 (100 nM) treatment for 72 hours, measured using cell viability assays, and the ratio of the constitutive ECAR to OCR.

(J) CRC cells treated with DMSO or IM156 (15 μM) for 72 hours were subject to cell cycle progression analysis. Data shown are mean ± S.D. of results from 3 individual experiments, two-tailed Student's *t* test.

Fig. S2

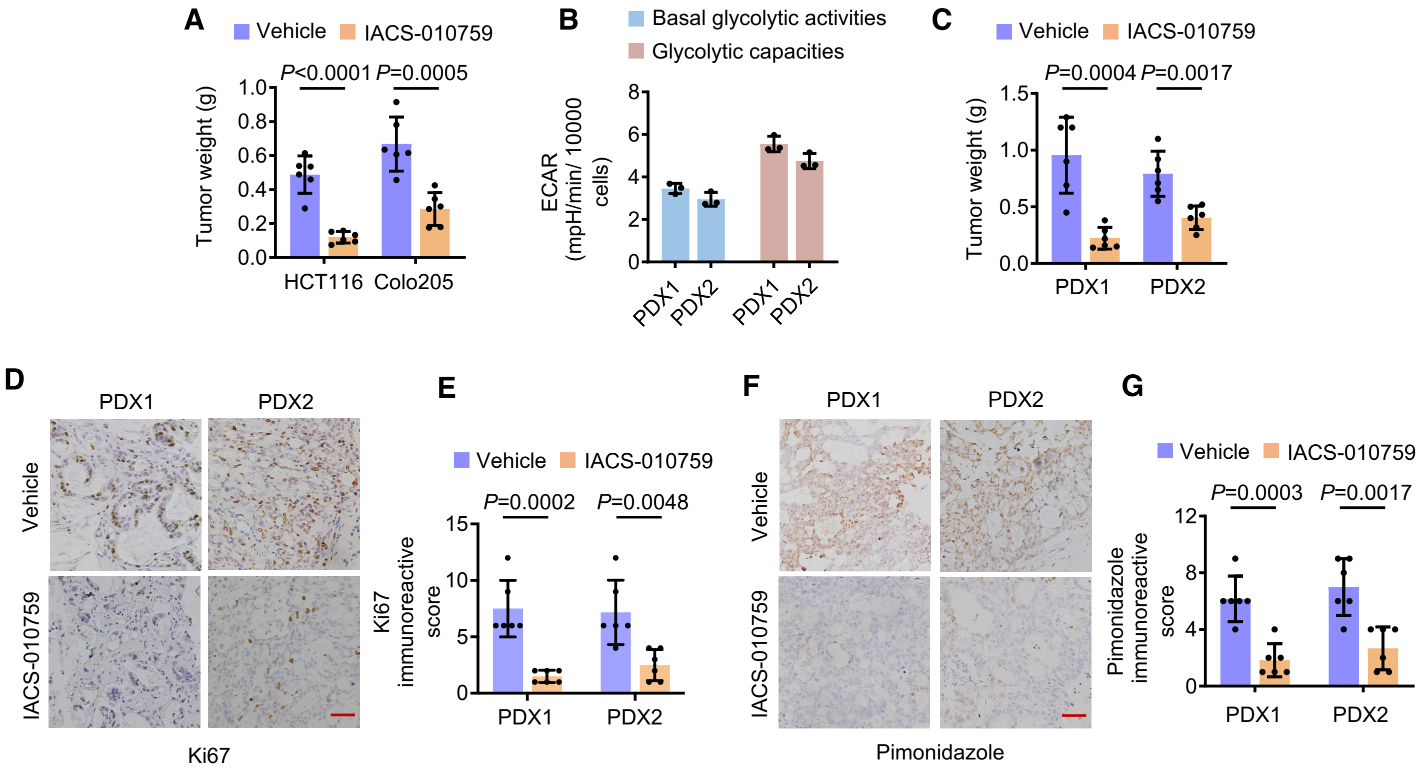

Fig. S2. OXPHOS inhibition retards CRC growth *in vivo*

(A) The weights of HCT116 and Colo205 xenografts harvested from NSG mice that were treated via oral gavage with the vehicle (0.5% methyl cellulose) or IACS-010759 (15 mg/kg). Data shown are mean ± S.D. of results from 6 mice per group, two-tailed Student's *t* test.

(B) The basal glycolytic activity and the glycolytic capacity of patient-derived CRC cells for the establishment of PDX1 and PDX2 were measured using Seahorse XF96 assays. Data shown are mean ± S.D. of results from 3 individual experiments.

C) The weights of PDX1 and PDX2 tumors harvested from NOD/SCID mice treated via oral gavage with the vehicle (0.5% methyl cellulose) or IACS-010759 (15 mg/kg). Data shown are mean ± S.D. of results from 6 mice per group, two-tailed Student's *t* test.

(D) Representative microphotographs of IHC staining of Ki67 on FFPE sections of PDX1 and PDX2 tumors harvested from NOD/SCID mice treated via oral gavage with the vehicle (0.5% methyl cellulose) or IACS-010759 (15 mg/kg). Scale bar: 50 μm.

(E) Quantitation of Ki67 staining as shown in **D.** Data shown are mean ± S.D. of results from 6 mice per group, two-tailed Student's *t* test.

(F) Representative microphotographs of IHC staining of pimonidazole on FFPE sections of PDX1 and PDX2 xenografts harvested from NOD/SCID mice treated via oral gavage with the vehicle (0.5% methyl cellulose) or IACS-010759 (15 mg/kg) that were administered via i.p with pimonidazole (60 mg/kg in saline) 3 hours before euthanasia. Scale bar: 50 μm.

(G) Quantitation of pimonidazole staining as shown in **F**. Data shown are mean ± S.D. of results from 6 mice per group, two-tailed Student's *t* test.

Fig. S3

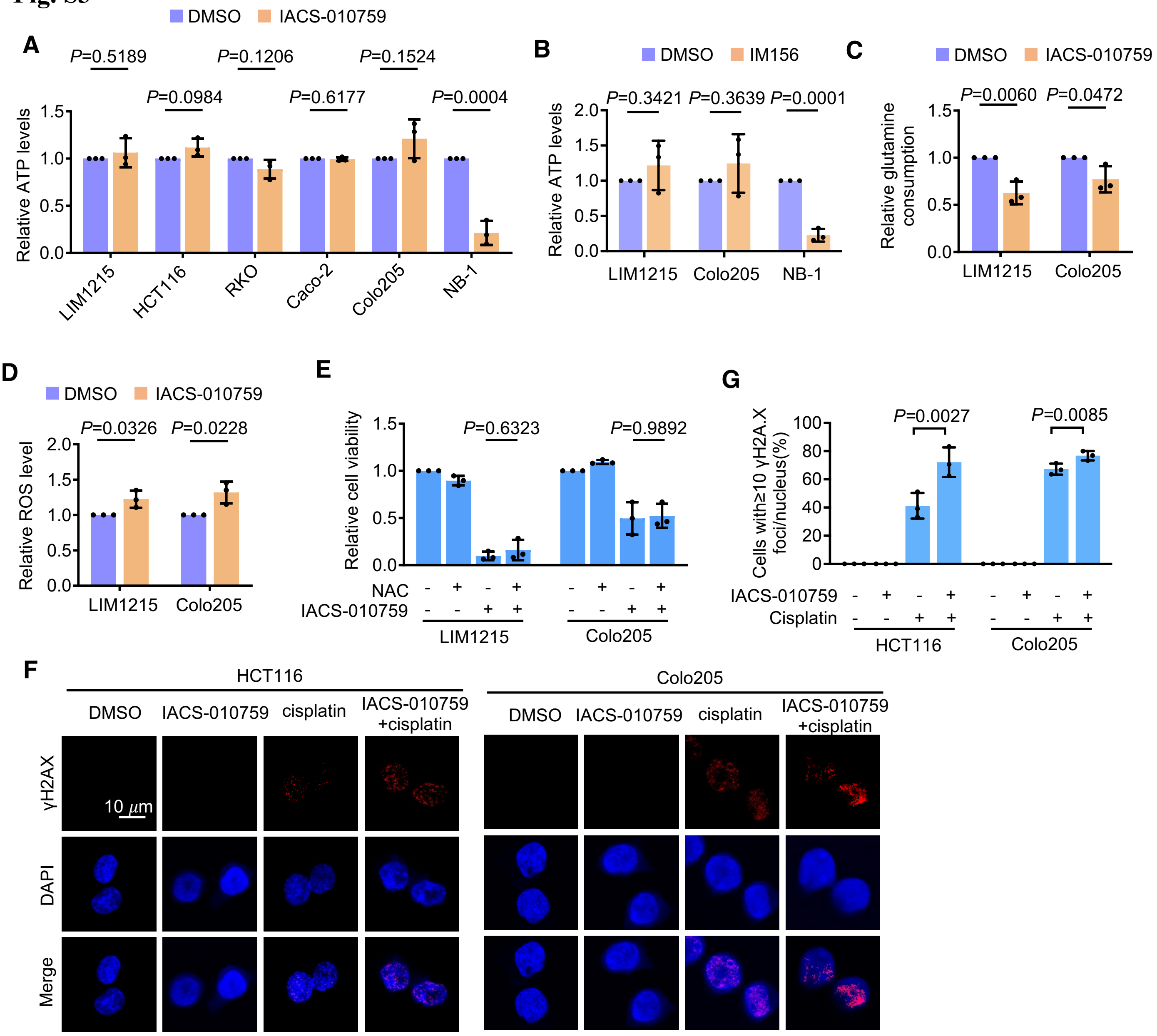

Fig. S3. OXPHOS inhibition does not cause devastating energy stress in CRC cells

(**A**) CRC cells and NB-1 neuroblastoma cells treated with DMSO or IACS-010759 (100 nM) for 24 hours were subjected to colorimetric ATP assays. Data shown are mean ± S.D. of results from 3 individual experiments, two-tailed Student's *t* test.

(**B**) LIM1215 and Colo205 CRC cells and NB-1 neuroblastoma cells treated with IM156 (15 μM) for 24 hours were subject to colorimetric ATP assays. Data shown are mean ± S.D. of results from 3 individual experiments, two-tailed Student's *t* test.

(**C**) LIM1215 and Colo205 cells were treated with the vehicle control (DMSO) or IACS-010759 (100 nM) for 24 hours. The culture media were subjected to colorimetric glutamine consumption assays. Data shown are mean ± S.D. of results from 3 individual experiments, two-tailed Student's *t* test.

(**D**) LIM1215 and Colo205 cells treated with the vehicle control (DMSO) or IACS-010759 (100 nM) for 48 hours were subjected to colorimetric ROS assays. Data shown are mean ± S.D. of results from 3 individual experiments, two-tailed Student's *t* test.

(**E**) LIM1215 and Colo205 cells treated with IACS-010759 (100 nM) in the absence or presence of NAC (2.5 mM) for 72 hours were subjected to cell viability assays. Data shown are mean ± S.D. of results from 3 individual experiments, one-way ANOVA followed by Tukey's multiple comparison test.

(**F**) HCT116 and Colo205 cells treated with IACS-010759 (100 nM) in the absence or presence of cisplatin (5 mM) for 24 hours were subjected to γH2A.X staining. Data shown are representative of 3 independent experiments.

(**G**) Quantitation of the percentage of cells with γH2A.X foci as shown in F. Data shown are mean ± S.D. of 3 independent experiments. One-way ANOVA followed by Tukey's multiple comparison.

Fig. S4

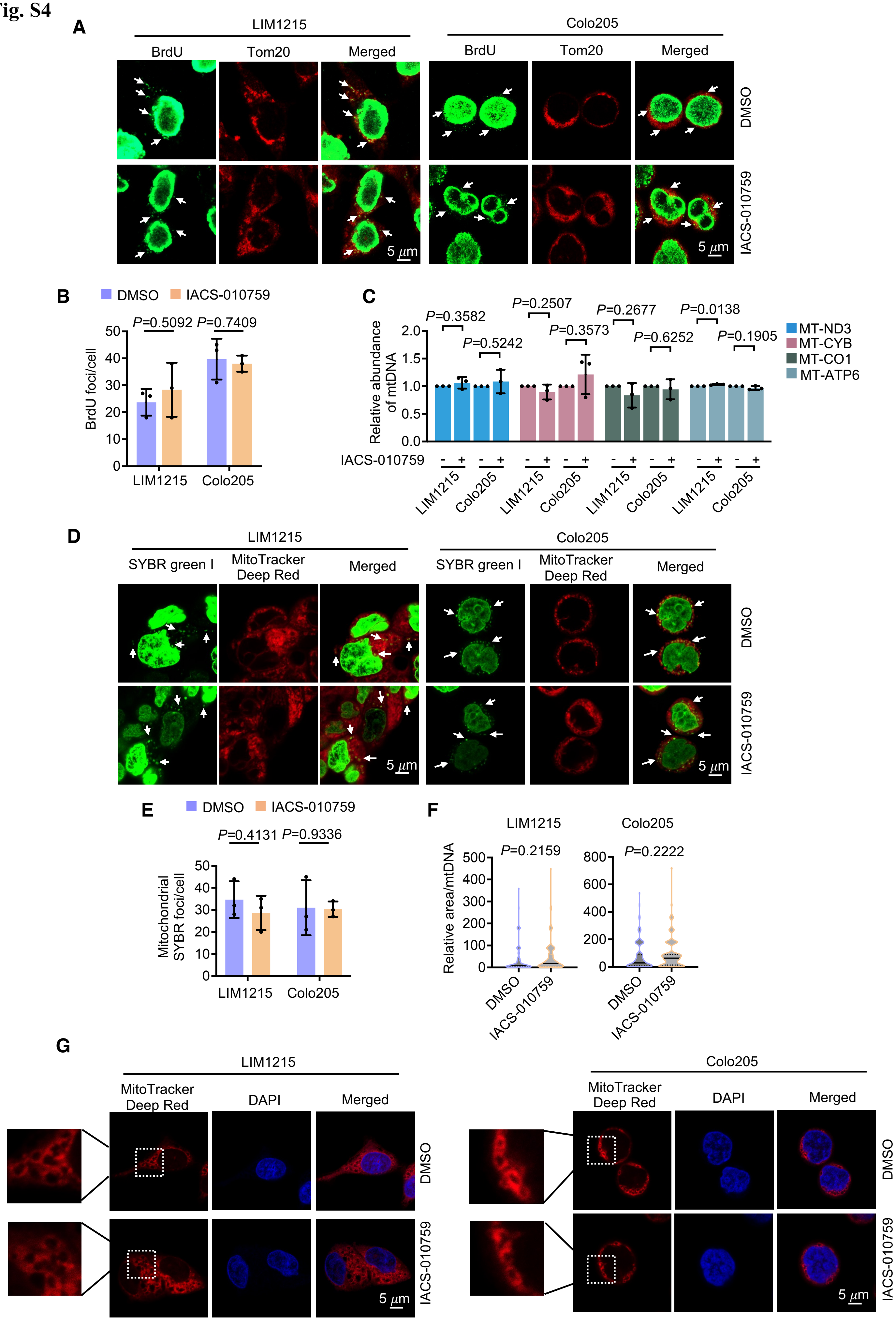

A
LIM1215
Colo205
BrdU
Tom20
Merged
DMSO
IACS-010759
5 μm
B
DMSO
IACS-010759
BrdU foci/cell
P=0.5092
P=0.7409
LIM1215
Colo205
C
Relative abundance of mtDNA
P=0.3582
P=0.5242
P=0.2507
P=0.3573
P=0.2677
P=0.6252
P=0.0138
P=0.1905
MT-ND3
MT-CYB
MT-CO1
MT-ATP6
IACS-010759
D
SYBR green I
MitoTracker Deep Red
Merged
E
Mitochondrial SYBR foci/cell
P=0.4131
P=0.9336
F
Relative area/mtDNA
P=0.2159
P=0.2222
G
DAPI

Fig. S4. OXPHOS inhibition does not cause mitochondrial DNA stress in CRC cells

(**A**) LIM1215 and Colo205 cells treated with DMSO or IACS-010759 (100 nM) for 24 hours were labelled with 50 μM 5-bromo-2′-deoxyuridine (BrdU) for 16 hours. After fixation, the cells were stained with anti-BrdU and Tom20 antibodies. Data shown are representative of 3 independent experiments.

(B) Quantitation of the BrdU foci in the mitochondria per cell as shown in **A** using imageJ software. Data shown are mean ± S.D. of results from 3 individual experiments, two-tailed Student's t test.

(C) LIM1215 and Colo205 cells treated with DMSO or IACS-010759 (100 nM) for 24 hours were subjected to genomic DNA extraction and qPCR assays. Data shown are mean ± S.D. of results from 3 individual experiments, two-tailed Student's t test.

(D) LIM1215 and Colo205 cells treated with DMSO or IACS-010759 (100 nM) for 24 hours were labelled with MitoTracker Deep Red and SYBR green I dye. Data shown are representative of 3 independent experiments.

(E and F) LIM1215 and Colo205 cells treated with as in **D** were subjected to measurement of Mitochondrial SYBR foci number per cell (**E**) and relative SYBR foci area per mtDNA using imageJ software. Data shown are mean ± S.D. of results from 3 individual experiments, two-tailed Student's t test.

(G) LIM1215 and Colo205 cells treated with DMSO or IACS-010759 (100 nM) for 24 hours were labelled with MitoTracker Deep Red. After fixation, cells were counterstained with DAPI. Data shown are representative of 3 independent experiments.

Fig. S5

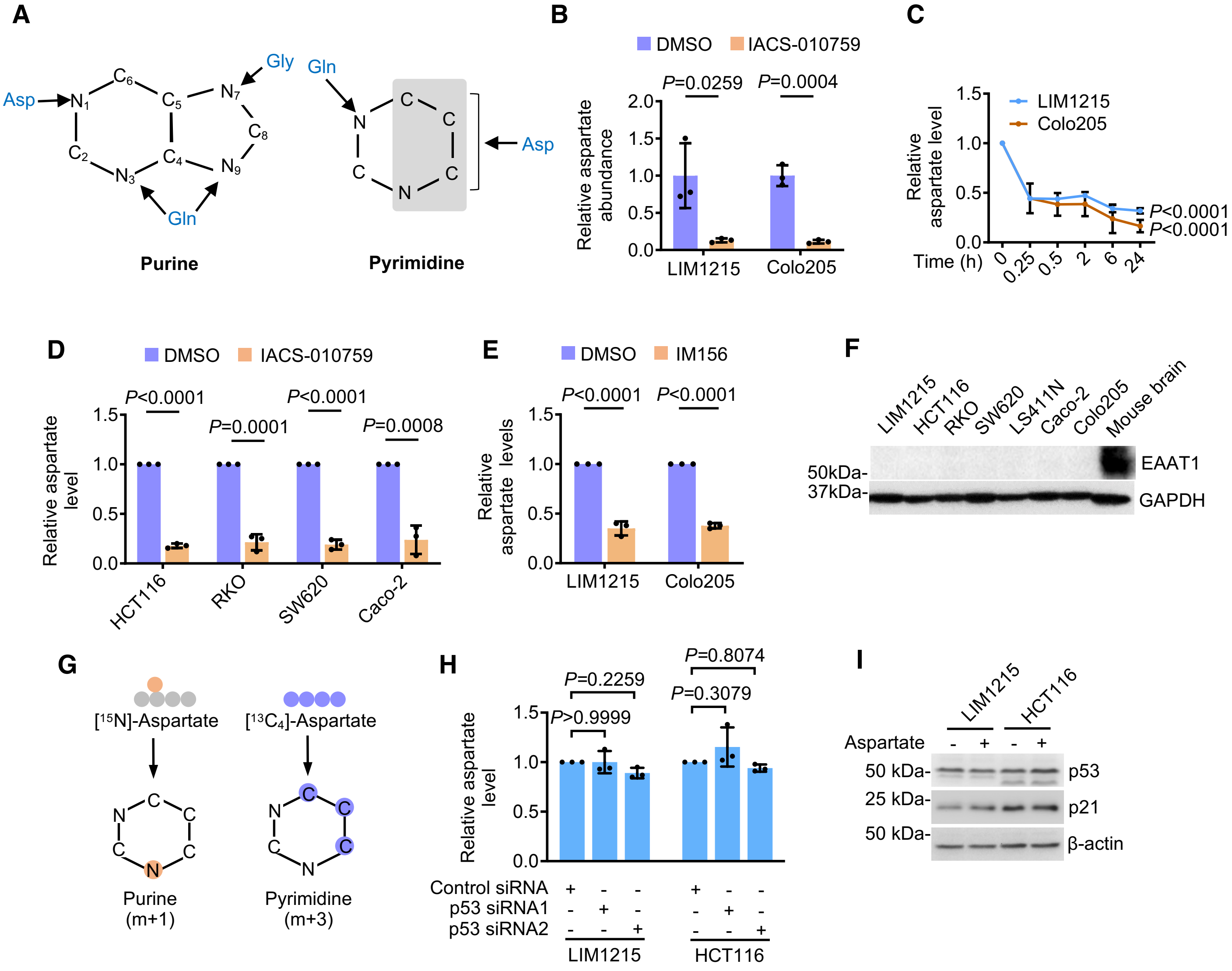

Fig. S5. An aspartate shortage contributes to nucleotide deficiencies upon OXPHOS inhibition

(A) A schematic illustration of aspartate donation of nitrogen and carbon atoms to purine and pyrimidine nucleobases, respectively. Asp, aspartate; Gln, glutamine; Gly, glycine.

(B) Extracted metabolites from LIM1215 and Colo205 cells treated with DMSO or IACS-010759 (100 nM) for 24 hours were subject to targeted metabolomics assays using LC-MS. Data shown are mean ± S.D. of results from 3 individual experiments, two-tailed Student's *t* test.

(C), LIM1215 and Colo205 cells with or without treatment with IACS-010759 (100 nM) for the indicated periods were subjected to colorimetric aspartate assays. Data shown are mean ± S.D. of results from 3 individual experiments, one-way ANOVA followed by Tukey's multiple comparison test.

(D) CRC cells treated with DMSO or IACS-010759 (100 nM) for 24 hours were subjected to colorimetric aspartate assays. Data shown are mean ± S.D. of results from 3 individual experiments, two-tailed Student's *t* test.

(E) LIM1215 and Colo205 cells were treated with DMSO or IM156 (15 μM) for 24 hours followed by colorimetric aspartate assays. Data shown are mean ± S.D. of results from 3 individual experiments, two-tailed Student's *t* test.

(F) Whole cell lysates from CRC cells and crude tissue extracts from mouse brain tissues were subjected to Western blot analysis. Data shown are representatives of 3 individual experiments.

(G) A schematic illustration of the isotope-tracing strategy with uniformly labelled [^{15}N]- or [^{13}C]-aspartate.

(H) Whole cell lysates from LIM1215 and HCT116 cells transfected with the control siRNA, p53 siRNA1, or p53 siRNA2 were subjected to colorimetric aspartate assays. Data shown are mean ± S.D. of results from 3 individual experiments, one-way ANOVA followed by Tukey's multiple comparison test.

(I) LIM1215 and HCT116 cells with or without treatment with 10 mM aspartate for 24 hours were subjected to Western blot analysis. Data shown are representatives of 3 individual experiments.

Fig. S6

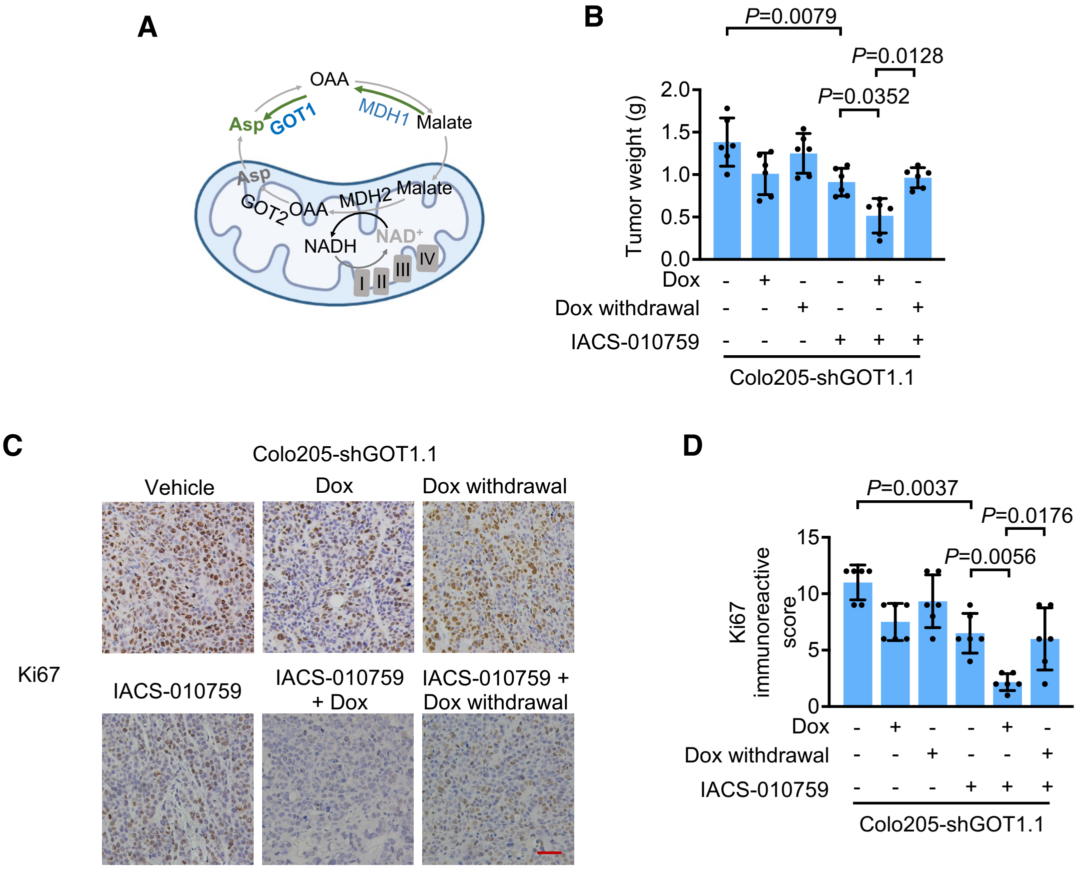

Fig. S6. Targeting GOT1 sensitizes CRC cells to the OXPHOS inhibition

(A) A schematic illustration of cytosolic aspartate synthesis catalyzed by GOT1 in cells when mitochondrial aspartate synthesis is blocked.

(B) The weights of Colo205 xenografts carrying inducible GOT1 shRNA1 harvested from NOD/SCID mice treated via oral gavage with the vehicle (0.5% methyl cellulose) or IACS-010759 (10 mg/kg) with or without co-treatment with Dox (2 mg/ml supplemented with 10 mg/ml sucrose in drinking water) and the cessation of Dox treatment. Data shown are mean ± S.D. of results from 6 mice per group, one-way ANOVA followed by Tukey's multiple comparison test.

(C) Representative microphotographs of IHC staining of Ki67 on FFPE sections of xenografts of Colo205 cells with inducible GOT1 shRNA1 harvested from mice treated as described in B. Data shown are representatives of 6 mice per group. Scale bar: 50 μm.

(D) Quantitation of Ki67 staining as shown in **C.** Data shown are mean ± S.D. of results from 6 mice per group, one-way ANOVA followed by Tukey's multiple comparison test.

Table S1. List of antibodies

Antibody (Ab)	Catalogue No.	Company	Dilution
AMPK Rabbit mAb[a]	2603	Cell Signalling Technology	1:1000
Phospho-AMPK (Thr172) Rabbit mAb	2531	Cell Signalling Technology	1:1000
β-actin Mouse mAb	sc-47778	Santa Cruz Biotechnology	1:2500
EAAT1 Rabbit mAb	5685	Cell Signalling Technology	1:1000
GAPDH Mouse mAb	sc-32233	Santa Cruz Biotechnology	1:2500
GOT1 Rabbit mAb	34423S	Cell Signalling Technology	1:1000
p53 Mouse mAb	sc-126	Santa Cruz Biotechnology	1:800
p21 Rabbit mAb	2947S	Cell Signalling Technology	1:1000
Ki-67 Rabbit mAb	9129S	Cell Signalling Technology	1:400
Anti-pimonidazole Rabbit pAb[b]	PAb2627	Hypoxyprobe	1:200
Iodouridine Mouse mAb	347580	BD Biosciences (Franklin Lakes, NJ)	1:500
Chlorouridine Rat mAb	ab6326	Abcam (Waltham, BOS)	1:300
Phospho-Histone H2A.X (Ser139) (D7T2V) Mouse mAb	80312S	Cell Signalling Technology	1:100
Tom20	42406S	Cell Signalling Technology	1:100
Anti-mouse IgG (H+L), F(ab')2 Fragment (Alexa Fluor® 594 Conjugate)	8890S	Cell Signalling Technology	1:500
Anti-rabbit IgG (H+L), F(ab')2 Fragment (Alexa Fluor® 594 Conjugate)	8889S	Cell Signalling Technology	1:100
Goat anti-Rat IgG (H+L) Alexa Fluor™ 488	A11006	Thermo Fisher Scientific	1:300
Goat Anti-Mouse IgG(H+L)-HRP Conjugate	1706516	Bio-Rad Laboratories	1:2500
Goat Anti-Rabbit IgG (H+L)-HRP Conjugate	1706515	Bio-Rad Laboratories	1:2500
Goat-anti-rabbit IgG-HRP	sc-2004	Santa Cruz Biotechnology	1:2500
Goat-anti-mouse IgG-HRP	sc-2005	Santa Cruz Biotechnology	1:2500

a: monoclonal antibody; b: polyclonal antibody

Table S2. List of chemicals

Reagent	Catalogue No.	Company
IACS-010759	25867	Cayman chemical
IM156	S9604	Selleckchem
2-Deoxy-D-glucose	D6134	Sigma-Aldrich
L-Aspartic acid	A7219	Sigma-Aldrich
Adenine	A8626	Sigma-Aldrich
Guanine	G11950	Sigma-Aldrich
Uridine	U3003	Sigma-Aldrich
5-Chloro-2'-deoxyuridine	C6891	Sigma-Aldrich
5-Iodo-2'-deoxyuridine	I7125	Sigma-Aldrich
5-Bromo-2′-deoxyuridine	B5002	Sigma-Aldrich
Pimonidazole Hydrochloride	HP-1000mg	Hydroxyprobe
Doxycycline	D9891	Sigma-Aldrich
Propidium iodide	P4170	Sigma-Aldrich
L-Aspartic acd-$^{13}C_4$	604852	Merck
L-Aspartic acd-^{15}N	332135	Sigma-Aldrich
Methyl cellulose	M7027	Sigma-Aldrich
Tris hydrochloride	93363	Sigma-Aldrich
Sodium chloride	S3014	Sigma-Aldrich
Calcium chloride	C5670	Sigma-Aldrich
Magnesium chloride	M8266	Sigma-Aldrich
Sodium Azide	S2002	Sigma-Aldrich
Triton X-100	93443	Sigma-Aldrich
SYBR™ Green I Nucleic Acid Gel Stain, 10,000X concentrate in DMSO	S7563	Thermo Fisher
MitoTracker™ Deep Red FM	M22426	Thermo Fisher

Table S3. siRNA, shRNA sequences

Target	Sequence
**GOT1**	siRNA.1/shRNA.1: CUCCUGAGUUCUCCAUUGU<br>siRNA.2/shRNA.2: GCGGAUUACUUGGUCCAAU
**p53**	siRNA.1: CGGCGCACAGAGGAAGAGAAUCUC<br>siRNA.2: GACUCCAGUGGUAAUCUAC
**Negative control**	siRNA/shRNA: UUCUCCGAACGUGUCACGU

Table S4. Common gene mutations in a panel of CRC cell lines.

Genes	LIM1215	HCT116	RKO	SW620	LS411N	Caco-2	Colo205
BRAF	WT[a]	WT	V600E	WT	V600E	WT	V600E
KRAS	WT	G13D	WT	G12V	WT	WT	WT
NRAS	WT	WT	WT	WT	WT	WT	WT
PIK3CA	WT	H1047R	H1047R	WT	WT	WT	WT
TP53	WT	WT	WT	R273H, P309S	Y126*	C135F, E204*	Y107fs; Y103fs
APC	V1804D	V1804D	WT	Q1338*	E1554fs, Q789*	Q1367*	V1804D
***MYC* copy number gain**	-	+	+	+	-	-	+

a: wildtype.

Table S5. qPCR primer sequences

Target	Sequence
MT-CYB	Forward: ATCACTCGAGACGTAAATTATGGCT<br>Reverse: TGAACTAGGTCTGTCCCAATGTATG
MT-COI	Forward: GACGTAGACACACGAGCATATTTCA<br>Reverse: AGGACATAGTGGAAGTGAGCTACAAC
MT-ND3	Forward: CCCTACCATGAGCCCTACAAACAA<br>Reverse: AGTCACTCATAGGCCAGACTTAGG
MT-ATP6	Forward: TAGCCATACACAACACTAAAGGACGA<br>Reverse: GGGCATTTTTAATCTTAGAGCGAAA
