# Supplementary figures and images for: DNA replication stress underpins the vulnerability to oxidative phosphorylation inhibition in colorectal cancer
Source: Cell Death Dis. 2025 Jan 14;16(1):16. doi: 10.1038/s41419-025-07334-4 (PMC11733219; doi:10.1038/s41419-025-07334-4)

**Figure 3B**

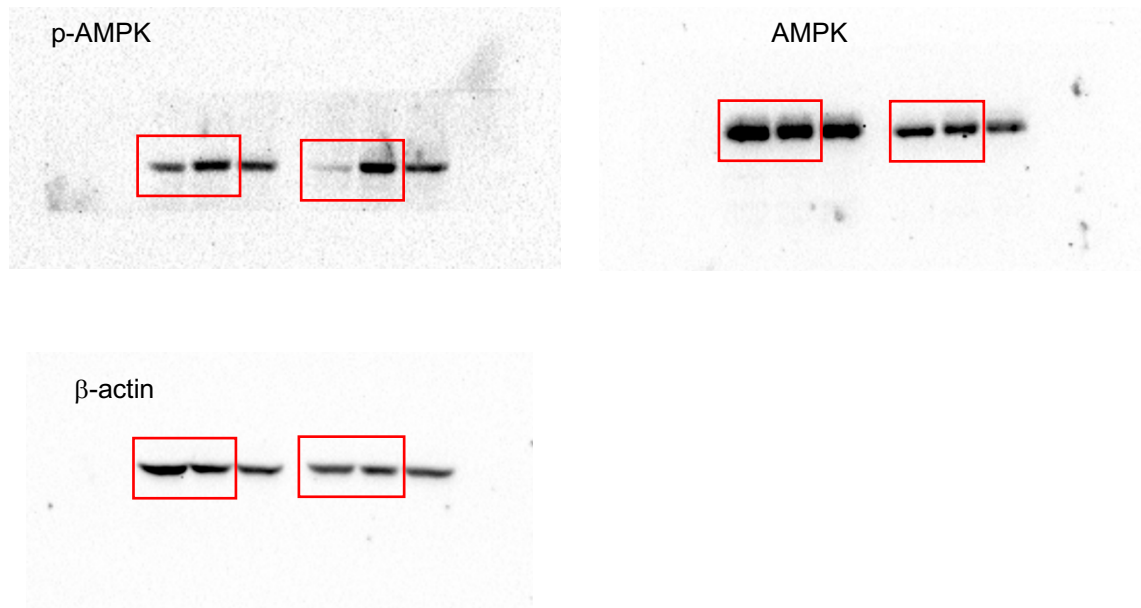

**Figure 4D**

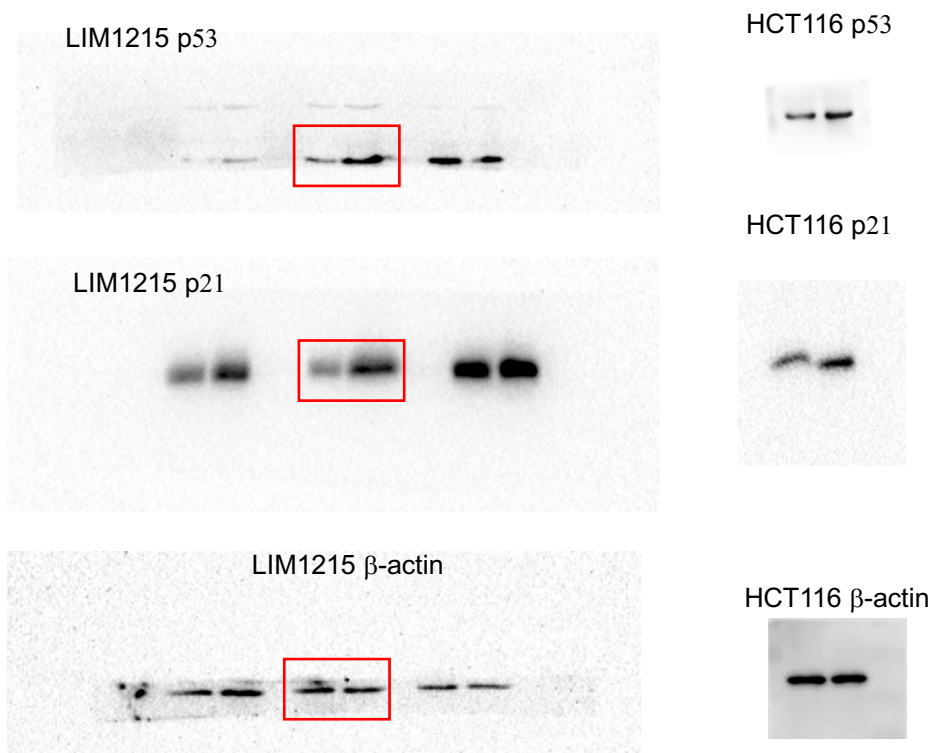

Figure 4E

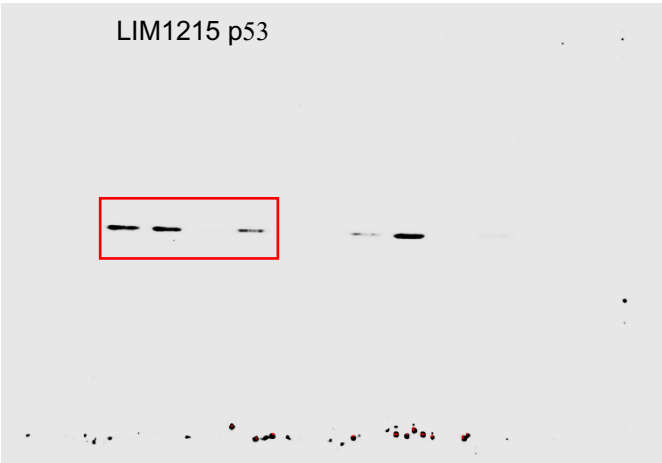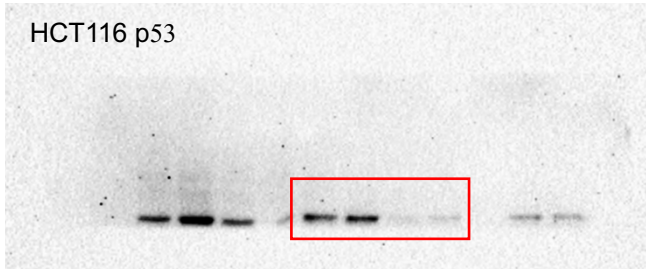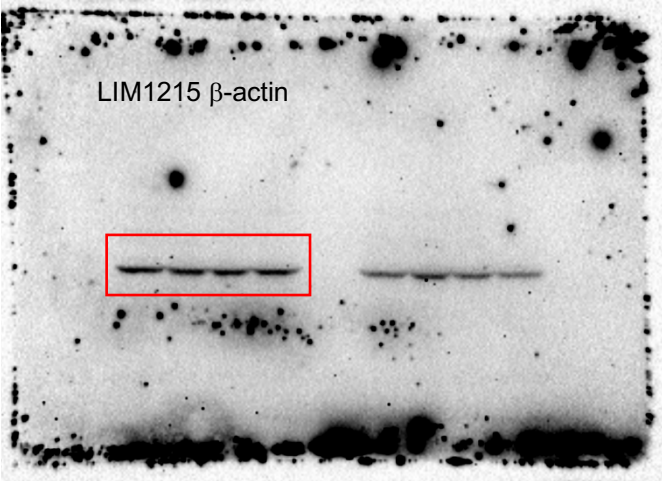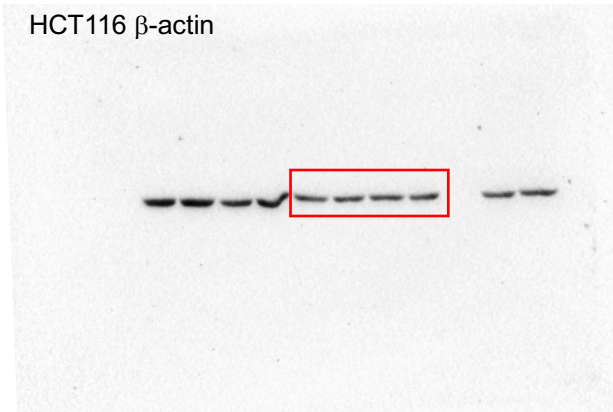

**Figure 6A**

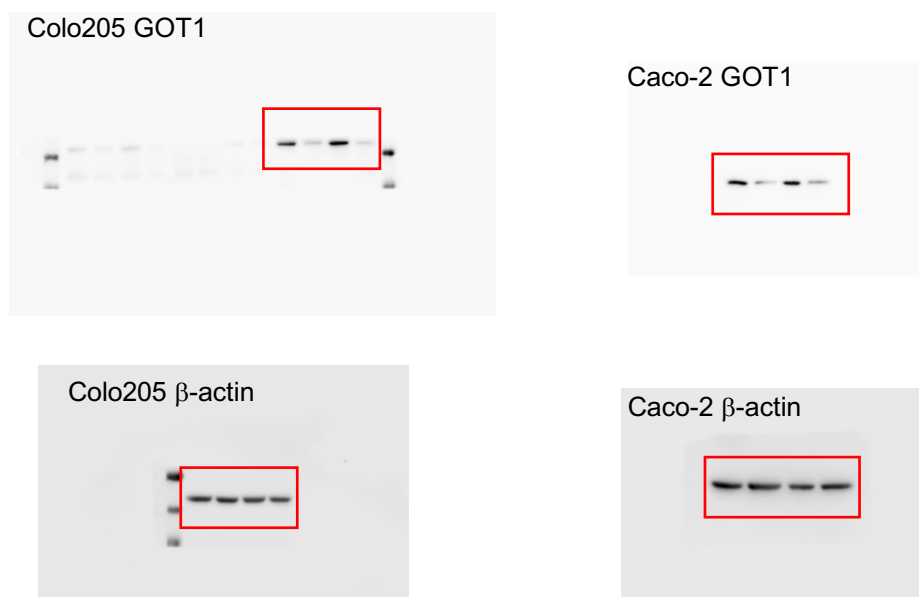

**Figure 6G**

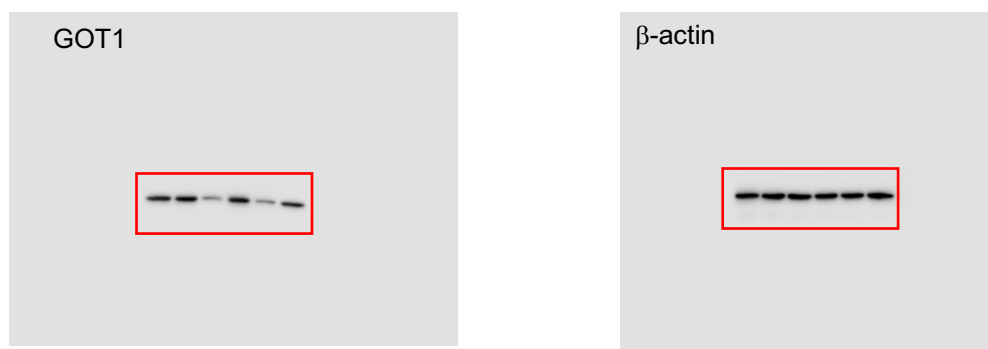

**Figure S5F**

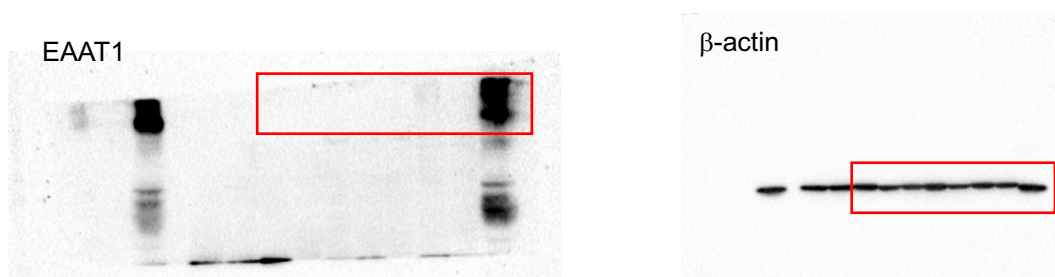

**Figure S5I**

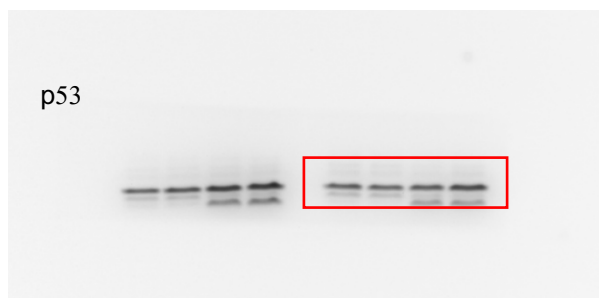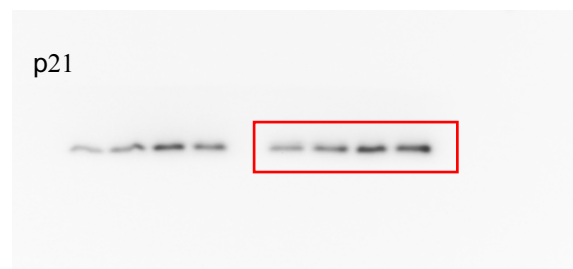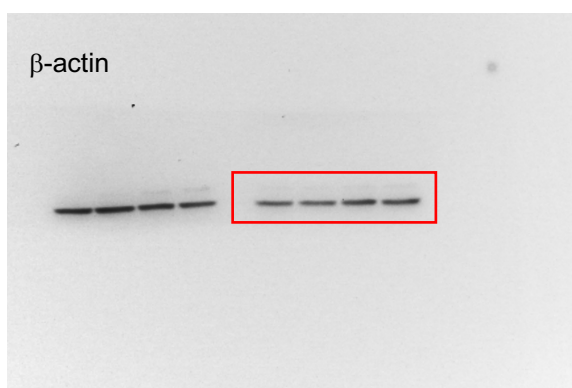

Supplement: Supplementary file 2 — Uncropped Western Blots [file 41419_2025_7334_MOESM2_ESM.pdf]
